# Evidence for PII with NAGK interaction that regulates Arg synthesis in the microalga *Myrmecia incisa* in response to nitrogen starvation

**DOI:** 10.1038/s41598-017-16644-3

**Published:** 2017-11-24

**Authors:** Yan Li, Wei Liu, Li-Ping Sun, Zhi-Gang Zhou

**Affiliations:** 1 Key Laboratory of Exploration and Utilization of Aquatic Genetic Resources Conferred by Ministry of Education, Shanghai Ocean University, Shanghai, 201306 China; 2 National Demonstration Center for the Experimental Teaching of Fisheries Science, Shanghai Ocean University, Shanghai, 201306 China; 3 International Research Center for Marine Biosciences Conferred by Ministry of Science and Technology, Shanghai Ocean University, Shanghai, 201306 China

## Abstract

To understand why most eukaryotic microalgae accumulate lipids during nitrogen starvation stress, a gene, *MiglnB*, encoding PII, a signal transduction protein, was cloned from the arachidonic acid-rich microalga *Myrmecia incisa* Reisigl. Similarly to its homologues, MiPII contains three conserved T-, B-, and C-loops. In the presence of abundant Mg^2+^, ATP, and Gln, MiPII upregulates Arg biosynthesis by interacting with the rate-limiting enzyme, MiNAGK, as evidenced by yeast two-hybrid, co-immunoprecipitation assays, and kinetics analysis of enzyme-catalyzed reactions. However, this interaction of MiPII with MiNAGK is reversed by addition of 2-oxoglutarate (2-OG). Moreover, this interaction is present in the chloroplasts of *M*. *incisa*, as illustrated cytologically by both immunoelectron microscopy and agroinfiltration of *Nicotiana benthamiana* leaves to determine the subcellular localization of MiPII with MiNAGK. During the process of nitrogen starvation, soluble Arg levels in *M*. *incisa* are modulated by a change in MiNAGK enzymatic activity, both of which are significantly correlated (*r* = 0.854). A model for the manipulation of Arg biosynthesis via MiPII in *M*. *incisa* chloroplasts in response to nitrogen starvation is proposed. The ATP and 2-OG saved from Arg biosynthesis is thus suggested to facilitate the accumulation of fatty acids and triacylglycerol in *M*. *incisa* during exposure to nitrogen starvation.

## Introduction

Nitrogen (N) is an essential nutrient that is required for the growth and development of higher plants and microalgae. In freshwater ecosystems, nitrogen is often obtained from rainwater leaching, especially in crop fields^1^. When microalgae grow abundantly under favorable conditions, they experience nitrogen deficiency and adapt physiologically to the changing nitrogen concentrations in water^2^. Nitrogen starvation results in diminished levels of amino acids, nucleic acids, proteins, nucleotides, and coenzymes in microalgae, because it is a necessary constitutional element of these compounds^3^. As a result, microalgae can allocate photosynthetic products and energy to the production of N-free compounds^3–5^. For example, *Chlamydomonas reinhardtii* ^6^ and *Nannochloropsis gaditana* ^7^ have been reported to be able to accumulate triacylglycerols (TAGs) during nitrogen starvation.

Similarly to *Chlamydomonas*, *Nannochloropsis*, and several other reported eukaryotic microalgae^3,4,8,9^, *Myrmecia incisa* Reisigl, a green coccoid microalga that has been characterized as having a high content of arachidonic acid (ArA, 20:4ω6), of 7% ArA by dry weight (DW), especially when this microalga is cultured under nitrogen starvation stress, whereas the protein level has been found to decrease from 23% to 14%^10^. This finding indicates that nitrogen starvation leads to a cellular imbalance in the carbon-to-nitrogen ratio (C/N), thus blocking protein synthesis but stimulating the accumulation of ArA. On the basis of previous reports^11–16^ showing that GlnB-type PII, a signal transduction protein encoded by *glnB*, generally functions as a C/N balance sensor in bacteria, archaea, and plants, we speculated that PII protein might link nitrogen metabolism and fatty acid synthesis in *M*. *incisa*.

The interaction between PII protein and *N*-acetyl-L-glutamate kinase (NAGK) or ATP:*N*-acetyl-L-glutamate 5-phosphotransferase (EC 2.7.2.8), the product of *argB*, has been demonstrated in rice^17^ and *Arabidopsis thaliana* ^18–20^, as well as in cyanobacteria^14,21^. NAGK catalyzes the rate-limiting step in arginine (Arg) biosynthesis, converting *N*-acetyl L-glutamate into *N*-acetyl L-glutamyl 5-phosphate^22,23^, and its activity is modulated by PII via the protein-protein interaction between them^24^. Under conditions of low levels of 2-oxoglutarate (2-OG) or α-ketoglutarate, the GlnB-type PII forms a complex with NAGK, thus releasing the inhibition of Arg to NAGK and promoting NAGK activity. In contrast, direct evidence of this interaction of PII protein with NAGK is scarce in eukaryotic green microalgae, which are considered to be an important phylogenetic link between cyanobacteria and higher plants.

In addition to NAGK, PII protein interacts with other target proteins, for example, biotin carboxyl carrier protein (BCCP), a subunit of acetyl-CoA carboxylase (ACCase, EC 6.4.1.2), to regulate its functions. A recent survey has suggested that BCCP interacts with PII in *Arabidopsis thaliana* ^25^, *Synechocystis* sp. PCC 6803^26^, *Escherichia coli*, and *Azospirillum brasiliense* ^27,28^. ACCase catalyzes the first and committed step, the carboxylation of acetyl-CoA to malonyl-CoA, of fatty acid synthesis^29^. In the green microalgae *Chlamydomonas reinhardtii* and *Chlorella variabilis* NC64A, a single GlnB-type PII homologue has been characterized by Ermilova *et al*.^30^ and Minaeva and Ermilova^31^, respectively. Recently, Zalutskaya *et al*.^32^ have found that knockdown of this type PII by artificial microRNA results in an over-accumulation of lipid bodies and an increase in the total TAG level in *Chlamydomonas reinhardtii*. This phenomenon has been confirmed in a PII mutant strain of *Synechocystis* sp. PCC 6803^26^. Therefore, it is reasonable to infer that PII protein might inhibit fatty acid synthesis in *Chlamydomonas* and other microalgae by interacting with ACCase to decrease this enzyme activity in the same manner as in both the higher plant *Arabidopsis* ^25^ and bacteria including cyanobacteria^26–28^. However, direct evidence for the interaction between PII protein and BCCP in these microalgae is apparently still required.

To test the above-mentioned hypothesis that the PII signal transduction protein links nitrogen metabolism and fatty acid synthesis by interacting with NAGK or ACCase in *M*. *incisa*, we cloned and characterized the genes encoding GlnB-type PII, NAGK, and BCCP from this ArA-rich microalga. The interaction between *M*. *incisa* PII protein and NAGK was examined by yeast two-hybrid assays, and it was confirmed by an *in vitro* co-immunoprecipitation assay after the preparation of recombinant PII and NAGK and subsequently the PII polyclonal antibody. However, the expected relationship between PII protein and BCCP was not detected. The subcellular localization of PII protein and NAGK was determined by immunoelectron microscopy and agroinfiltration of *Nicotiana benthamiana* leaves, respectively. The dissociation of PII from the interacting NAGK could block Arg synthesis in *M*. *incisa* in response to nitrogen starvation, on the basis of co-immunoprecipitation assay, kinetics analysis of enzyme-catalyzed reactions and the relationship between soluble Arg levels and NAGK activity. The 2-OG and ATP saved from Arg biosynthesis has been proposed to facilitate the reported lipid synthesis and accumulation in *M*. *incisa* ^10,33^ and several other microalgae^4,6,7,9^. The present study provides novel insights into one explanation for why these eukaryotic microalgae accumulate lipid or starch during nitrogen starvation stress.

## Results

### Characterization of the *MiglnB* gene and its deduced protein

**Figure 1:**
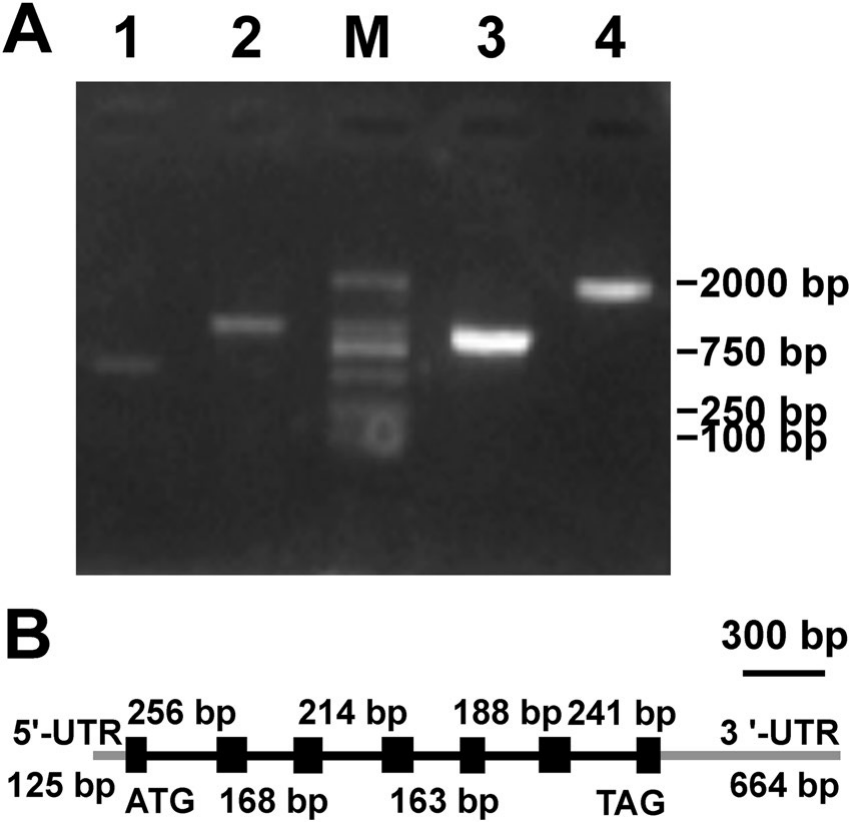
Electrophoresis patterns of PCR products (**A**) and gene structure (**B**) of *MiglnB* cloned from *Myrmecia incisa*. Lane 1, 5′-RACE product; Lane 2, 3′-RACE product; Lane 3, ORF of *MiglnB* using RT-PCR; Lane 4, genomic DNA product of *MiglnB*; and Lane M, 2000-bp DNA marker. Exons and introns are indicated by a black box and line, respectively, and the UTR is indicated by a grey line.

From the transcriptome database^34^ in *Myrmecia incisa*, a 627-bp Contig21987-10 was found to have 47% identity to the PII-coding sequence (*GLB1*, GenBank accession No. EDO96407) of *Chlamydomonas reinhardtii*. On the basis of this contig sequence, 3 primers (5GSP1, 5GSP2, and 3GSP, Supplementary Table S1) were designed, and a gene, designated *MiglnB*, was cloned from *M*. *incisa* by using the 5′/3′ rapid amplification of cDNA ends (RACE) technique (Fig. 1A). After manual assembly and PCR re-amplification with newly designed primers (data not shown), the full-length cDNA sequence of *MiglnB* was 1,391 bp long and included a 125-bp 5′-untranslated region (UTR) and a 636-bp 3′-UTR with a typical poly-A tail. According to this cloned sequence, one pair of primers, PII-F and PII-R (Supplementary Table S1), was designed to clone the *MiglnB* genomic DNA sequence (Fig. 1A). The genomic sequence was 1,855 bp in length and interrupted by six introns (Fig. 1B), similarly to that in *Chlorella variabilis* NC64A^31^. The sizes of these introns ranged from 163 bp to 256 bp. Both the 5′- and 3′-ends of each intron contained splice sites that conformed to the GT-AG rule identified in nuclear genes.

The 630-bp open reading frame (ORF) of *MiglnB* encodes a protein, MiPII, which was predicted to consist of 209 amino acids (aa) with a calculated molecular mass of approximately 21 kD. The presence of a potential 52-aa chloroplast signal peptide (Supplementary Fig. S1) as predicted online using the ChloroP 1.1 Server, and the highest score for chloroplast (1.880) while comparing with those for mitochondrion (0.652) and secretion pathway (0.225) as computed online by PredAlgo Server, suggested that the mature MiPII protein might reside in chloroplasts. Amino acid sequence alignment (Supplementary Fig. S1) showed that MiPII had three functionally essential regions: T-loop, B-loop, and C-loop, which were generally involved in other PII proteins, as reviewed by Chellamuthu *et al*.^16^. The deduced amino acid sequence of MiPII was similar to its plant and cyanobacterial homologues, sharing 52% identity with its homologue from *Chlamydomonas reinhardtii* (GenBank accession No. EDO96407), 50% with that from *Synechocystis* sp. PCC 6803 **(**GenBank accession No. CAA66127), and 47% with that from *Arabidopsis thaliana* (GenBank accession No. NP_192099). A phylogenetic tree (Supplementary Fig. S2) inferred from MiPII and other homologues using the neighbor-joining method^35^ illustrated that the PII proteins from green algae containing *M*. *incisa*, *Chlamydomonas reinhardtii*, and *Micromonas pusilla* CCMP1545 (GenBank accession No. EEH52861) could be grouped into a sub-cluster that was close to the higher plant sub-cluster, with a support bootstrap value of 99%.

### Characterization of the *MiargB* and *MiaccB* genes

In the *M*. *incisa* transcriptome database^34^, a 1,103-bp Contig9491_6 was searched and showed 67.2% identity to *Polytomella parva* NAGK (GenBank accession No. ABH11021). According to this contig, a pair of primers, NAGK-F1 and NAGK-R1 (Supplementary Table S1), was designed to clone *MiargB* encoding *M*. *incisa* NAGK. After PCR amplification (Supplementary Fig. S3) and sequence confirmation, the ORF of *MiargB* was found to be 1,080 bp in length and to encode a 359-aa protein with a 59-aa chloroplast signal peptide (Supplementary Fig. S4). The amino acid sequence of MiNAGK shared 78% identity with *Chlorella variabilis* (GenBank accession No. XM_005844217), 77% with *Coccomyxa subellipsoidea* (GenBank accession No. XM_005651114), and 76% with *Chlamydomonas reinhardtii* (GenBank accession No. XM_001689409) NAGKs, thus indicating their conservation. The conserved sequence was observed in both PII signature binding box (PII-signature, BLOCKS Database accession number IPB002187A, http://www.blocks.fncrc.org/) and amino acid kinase domain [Protein Family Database (Pfam) accession number PF00696, http://www.sanger.ac.uk/Software/Pfam/] (Supplementary Fig. S4).

According to the searched 449-bp (Contig22797_3) and 632-bp (Contig10579_6) contigs, which shared 77.1% and 33.3% identity, respectively, with BCCP, the subunit of *Chlamydomonas reinhardtii* ACCase (GenBank accession Nos. XP_001700442 and XP_001690119, respectively), 5 primers (BCCP1-5, BCCP1-3, BCCP1-3N, BCCP2-F, and BCCP2-R, Supplementary Table S1) were designed to clone *MiaccB* from *M*. *incisa*. After PCR amplification (Supplementary Fig. S3), two genes, designated *MiaccB1* and *MiaccB2*, were assembled and then verified by PCR amplification by using re-designed primers (data not shown). The full-length cDNA of *MiaccB1* comprised a 44-bp 5′-UTR, a 524-bp 3′-UTR, and a 699-bp ORF, whereas the ORF of *MiaccB2* was 789 bp long. The deduced proteins, MiBCCP1 and MiBCCP2 encoded by *MiaccB1* and *MiaccB2*, respectively, were homologous to *Chlamydomonas reinhardtii* BCCP1 (GenBank accession No. EDO98131) and BCCP2 (GenBank accession No. EDP09857) with 60% and 53% identity, respectively. The identity between MiBCCP1 and MiBCCP2 was only 41%, thus suggesting that *M*. *incisa* contained at least had two isoforms of BCCP. A chloroplast signal peptide was predicted to be present in both MiBCCP1 and MiBCCP2, and it was composed of 45 residues in MiBCCP1 but 49 residues in MiBCCP2 (Supplementary Fig. S5). Although both had 3 domains, as illustrated in this multi-sequence alignment (Supplementary Fig. S5), the 232-aa MiBCCP1 had a biotin binding motif (194EAMKLMNEIE204), whereas the 263-aa MiBCCP2 lacked this motif, as noted by Cronan and Waldrop^29^ in the review of ACCases.

The cDNAs of these 4 genes, *MiglnB*, *MiargB*, *MiaccB1*, and *MiaccB2*, have been deposited in GenBank under accession Nos. KY849357, KY849358, KY849359, and KY849360, respectively, and the DNA sequence of *MiglnB* has been deposited under accession No. KY849361.

### Identification of proteins that interact with MiPII

**Figure 2:**
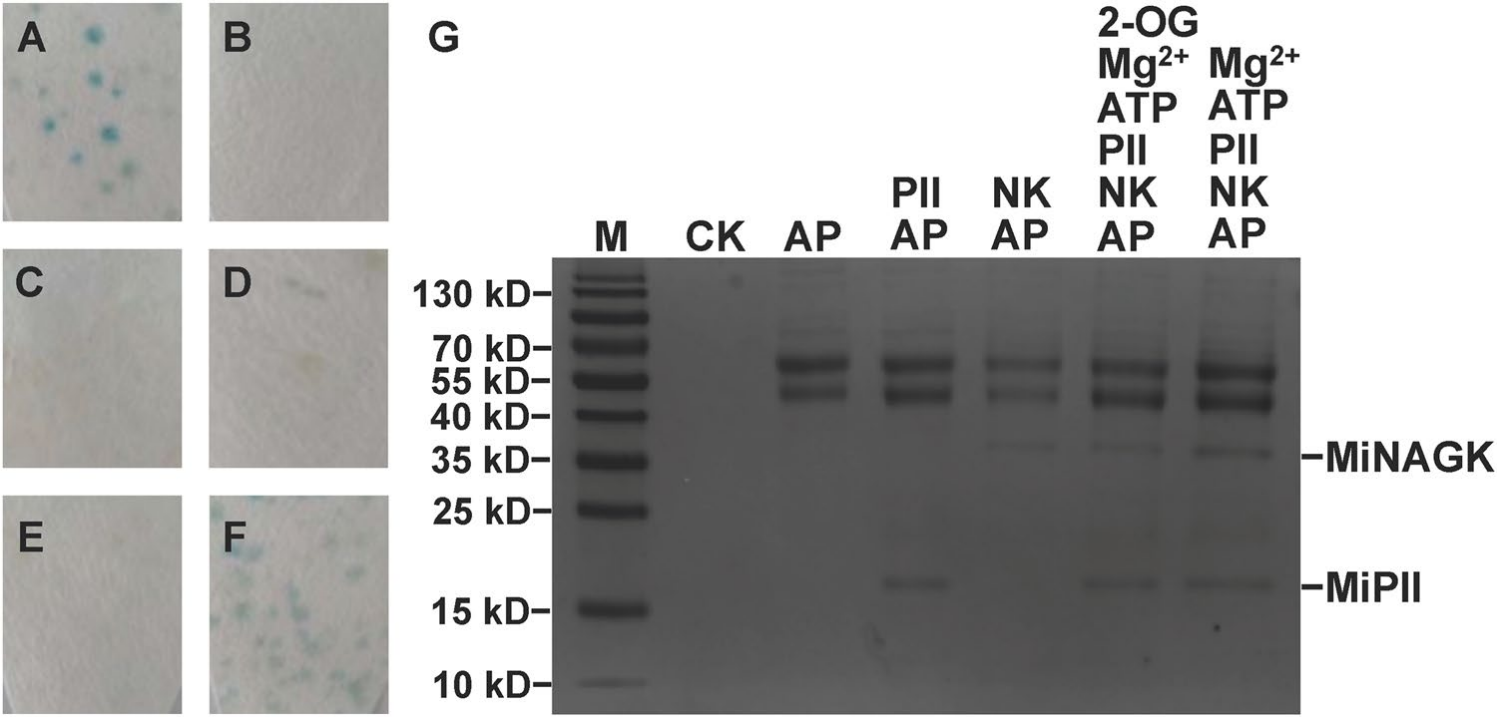
Protein-protein interaction between MiPII and MiNAGK as detected by yeast two-hybrid (from A through F) and co-immunoprecipitation (**G**) assays. Images A through E show yeast co-transformed with a pair of either pGBKT7-53 and pGADT7-T as a positive control (**A**) or pGBKT7-Lam and pGADT7-T as a negative control (**B**). From C through F iamges, yeast co-transformed with either pGBKT7-glnB only (**C**), pGBKT7-glnB and pGADT7-accB1 (**D**), pGBKT7-glnB and pGADT7-accB2 (**E**), or pGADT7-argB and pGBKT7-glnB (**F**). In Image G, 6 μL, 3 μL, and 5 μL of 1 M Mg^2+^, ATP, and 2-OG as marked above the panel, respectively, were added to 1 mL solution containing 200 μg and 400 μg recombinant MiPII and MiNAGK, respectively. The presence of MiNAGK bound to the Sepharose beads was subsequently analyzed by SDS-PAGE (15% acrylamide) and Coomassie blue R-250 staining. Lane M, protein molecular marker. AP, anti-MiPII polyclonal antibody. CK, only Sepharose as a control. NK, MiNAGK.

The yeast two-hybrid assay approach was used to identify *in vivo* protein-protein interaction between MiPII and MiNAGK, MiBCCP1, or MiBCCP2. The ORFs of *MiglnB*, *MiargB*, *MiaccB1*, and *MiaccB2* minus their corresponding nucleotide sequences of predicted signal peptides were cloned and ligated into vectors pGBKT7 and pGADT7 to generate the recombinants pGBKT7-glnB, pGADT7-argB, pGADT7-accB1, and pGADT7-accB2 (Supplementary Fig. S6). Each pair of vectors between pGBKT7-glnB and one of the others were co-transformed into yeast AH109, and the β-galactosidase activity of the transformed yeast was detected by using X-gal as the substrate. The yeast clone co-transformed with pGBKT7-glnB and pGADT7-argB turned blue (Fig. 2F), thus suggesting that MiPII should interact with MiNAGK in yeast cells. Unfortunately, the yeast clones carrying the vector pair of either pGBKT7-glnB and pGADT7-accB1 (Fig. 2D) or pGBKT7-glnB and pGADT7-accB2 lacked this color (Fig. 2E), thus suggesting that there might be no interaction between MiPII and MiBCCP1 or MiBCCP2.

To clarify the *in vitro* interaction of MiPII with MiNAGK, recombinant MiPII and MiNAGK proteins fused to the His-tag were expressed in *Escherichia coli*. The molecular masses of mature MiPII and MiNAGK minus signal peptide were predicted to be approximately 16.9 kD and 31.8 kD, respectively, but the recombinant MiPII and MiNAGK proteins were found to correspond to 21-kD and 36-kD bands (Supplementary Figs S7 and S8), respectively, because of the fused His-tag at their N-terminus. After purification using metal-chelate affinity chromatography with Ni-NTA resin, the purified recombinant MiPII protein was used to raise an antiserum against MiPII. In Western blot analysis, the total microalgal crude proteins immunologically reacted with the prepared anti-MiPII polyclonal antibody and revealed the presence of one unique band corresponding to the size of the computer-predicted mature MiPII (approximately 17 kD) (Supplementary Fig. S7, Lanes 12 and 13), thus suggesting that this antibody should be specific and reliable for subsequent experiments. In addition, the size of the mature MiPII as analyzed by Western blotting plus its putative 52-aa signal peptide was equal to the calculated molecular mass of MiPII precursor (21 kD), thus supporting the signal peptide prediction by using the ChloroP 1.1 Server.

For the *in vitro* co-immunoprecipitation assay, 1:2 (w/w) of the purified recombinant MiPII and MiNAGK were mixed in a solution with the other reagents as described below. After incubation for 1 h at room temperature, anti-MiPII polyclonal antibody was added, and proteins were pulled down with Staphylococcal protein A (SPA) according to the manufacturer’s instructions. The sodium dodecyl sulfate (SDS)-polyacrylamide gel electrophoresis (PAGE) pattern (Fig. 2G) of the precipitate revealed two bands of approximately 21 kD and 36 kD corresponding to the recombinant MiPII and MiNAGK (Supplementary Figs S7 and S8), respectively, in the solution containing Mg^2+^ and ATP. The appearance of a 36-kD band corresponding to MiNAGK in the lane NK + AP of Fig. 2G probably resulted from the immune reaction of His-Tag as co-expressed with the recombinant MiPII and MiNAGK. When 2-OG was added to the incubation solution, the intensity of the stained band for MiNAGK was approximately 0.65 times as much as that without addition of 2-OG (Fig. 2G) as estimated by ImageJ, thus suggesting lower content of MiNAGK in the precipitate. This co-immunoprecipitation assay therefore verified that MiNAGK interacted *in vitro* with MiPII in the presence of Mg^2+^ and ATP, but this interaction was antagonized by 2-OG.

### MiNAGK activity was enhanced by MiPII but inhibited by Arg or 2-OG

**Figure 3:**
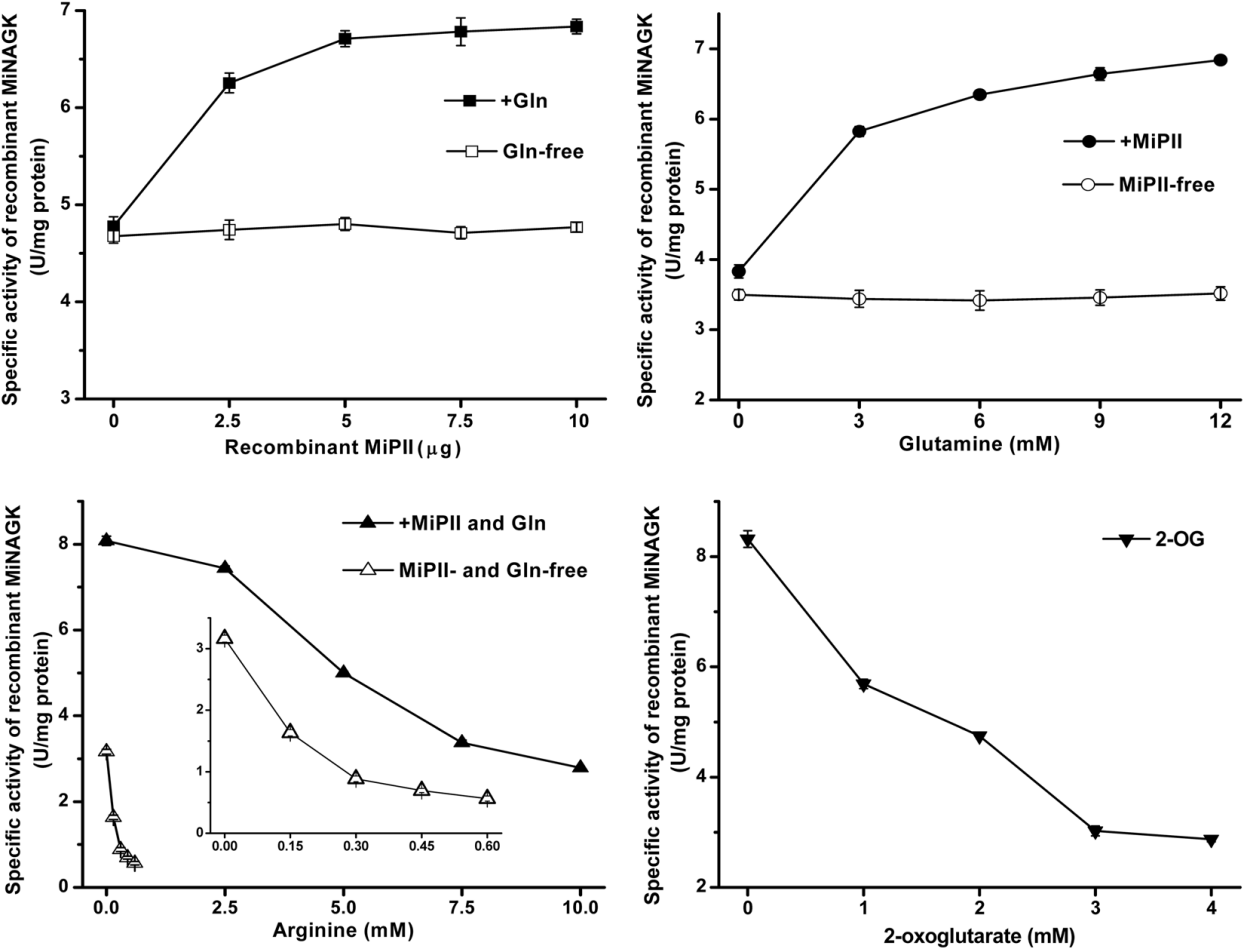
Effects of MiPII, Arg, 2-OG, or Gln on the activity of recombinant MiNAGK. The specific activity of the recombinant MiNAGK (5 μg in the reaction solution) was detected in the presence of recombinant MPII (10 μg) when it is needed. The inset in the lower left panels is the enlarged plot while MiPII and Gln are absent from the detection. The standard deviation from triplicate measurements is indicated by error bars.

To understand the effect of MiPII on MiNAGK via protein-protein interaction, the specific activity of the recombinant MiNAGK was estimated. When various levels of recombinant MiPII (from 0 through 10 μg) were added in the reaction solution while glutamine (Gln) was absent, the activity of MiNAGK showed little altered (Fig. 3, upper panels). In the presence of Gln (10 mM), however, its activity was enhanced by addition of recombinant MiPII, and the activity increased with the increase of MiPII levels, suggesting Gln was necessary for the enhancement of MiNAGK activity by MiPII. This finding was in good agreement with that the interaction of *Chlamydomonas* PII with NAGK was glutamine-dependent^36^. This enhancement was found to be dependent on Gln levels, and the activity got to the highest at 12 mM Gln in the reaction solution (Fig. 3, upper panels). Therefore, certain amounts of Gln (10 mM) and MiPII (10 μg) were used to explore the effects of Arg and 2-OG on the activity of MiNAGK.

This antagonistic effect of 2-OG on the MiNAGK activity was revealed by investigation with various levels of 2-OG. One mM of 2-OG in the reaction solution could make the specific activity of the recombinant MiNAGK drop by approximately 30% (Fig. 3, lower panels), and the activity of MiNAGK tended towards a decrease with the increase of 2-OG levels. Arg also had an inhibitory effect on the MiNAGK activity, and this inhibition was more obvious when MiPII and Gln were absent from the reaction solution (Fig. 3, lower panels). In the absence of MiPII and Gln, the half inhibition of the specific activity of MiNAGK was showed nearly at 0.14 mM Arg, while it was approximately 4.5 mM Arg when MiPII and Gln were present (Fig. 3, lower panels). These data suggested that MiPII and Gln could alleviate Arg inhibition of MiNAGK.

### Subcellular localization of MiPII and MiNAGK

**Figure 4:**
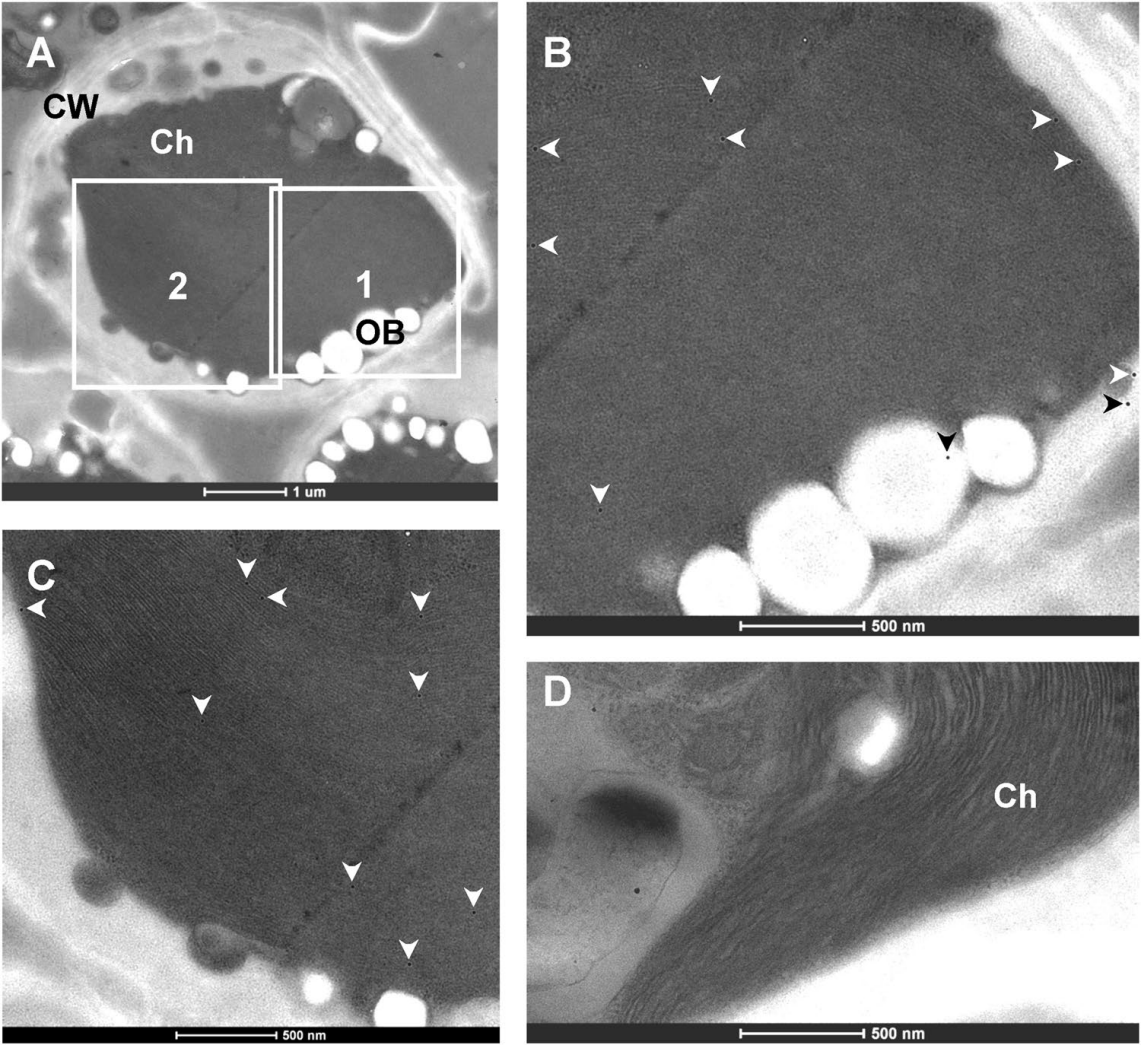
Transmission electron micrographs showing the immunogold labeling of *Myrmecia incisa* probed with the purified anti-MiPII polyclonal antibody. Ultrastructure of a whole cell of *Myrmecia incisa* (**A**–**C**) show enlarged images corresponding to the marked areas 1 and 2 in Image (**A**), revealing the distribution of the labeled immunogold particles as indicated by arrowheads. In Image (**D**), only anti-rabbit IgG conjugated to gold particles is applied to *M*. *incisa* cells as a control. Ch, chloroplast. CW, cell wall. OB, oil body.

Using the polyclonal antibody to MiPII prepared as described above, immunoelectron microscopy was performed to examine the subcellular localization of MiPII in *M*. *incisa*. Microalgal ultrathin sections incubated sequentially with the purified antibody to MiPII and the secondary antibody, anti-rabbit IgG conjugated to gold particles, were used to examine the ultrastructure of *M*. *incisa* and immunogold labeling signals under a transmission electron microscope. Approximately 83% of the immunogold particles labeled with the anti-MiPII polyclonal antibody were found decorating the microalgal chloroplasts or plastids, and the labeling appeared as either isolated or clustered particles (Fig. 4). The labeling density in chloroplasts was 7.3 particles/µm^2^ (n = 10), which was much higher than that in other regions of the microalgal cells (1.2 particles/µm^2^). This subcellular distribution of MiPII protein was highly significantly different (*P* = 0.001 < 0.01). MiPII was therefore thought to reside in microalgal chloroplasts, a result in good agreement with the subcellular localization prediction according to the presence of the chloroplast signal peptide (Supplementary Fig. S1).

**Figure 5:**
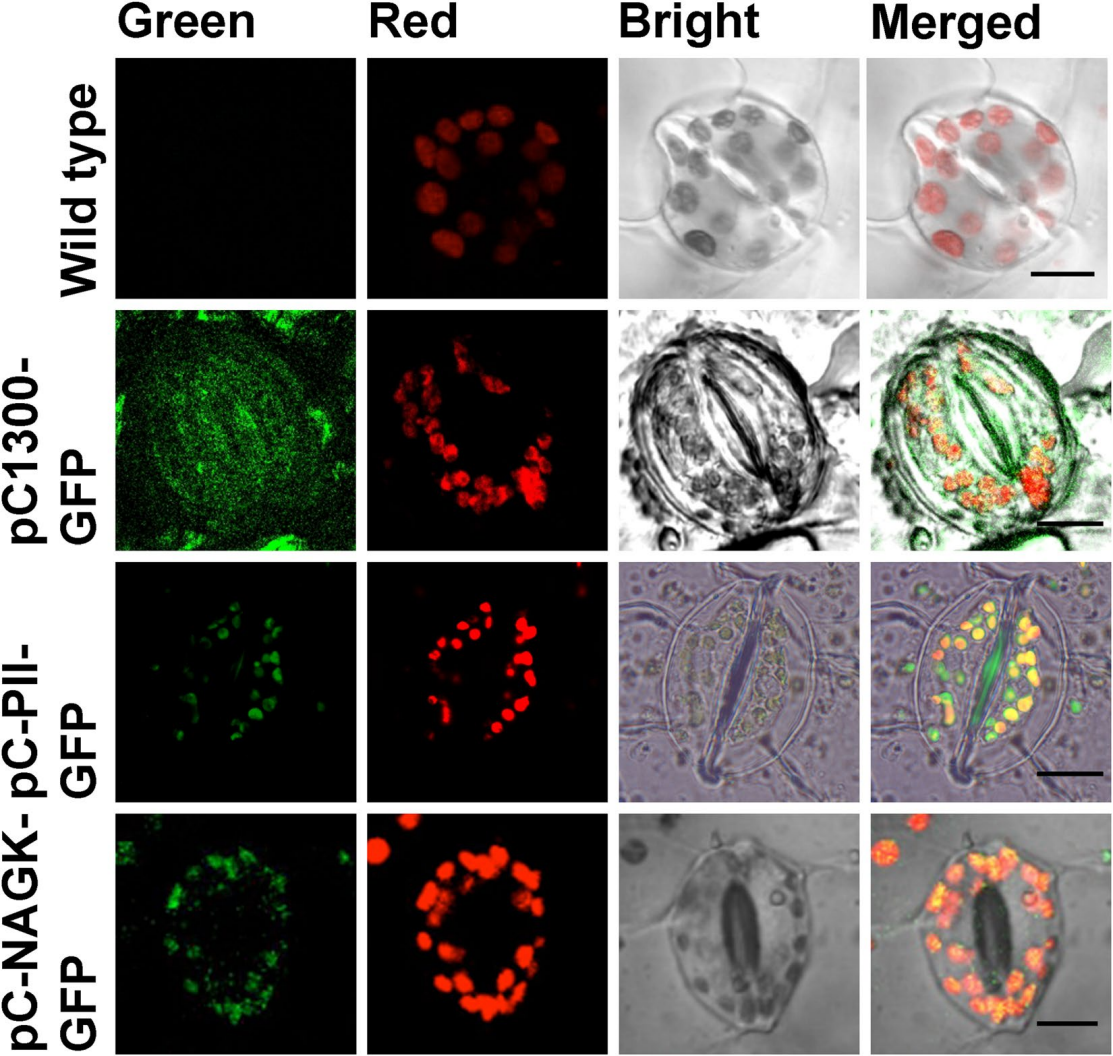
Confocal microscopic images showing the subcellular localization of MiNAGK and MiPII fused to GFP via agroinfiltration of *Nicotiana tabacum* leaves. A sequence encoding GFP was fused downstream of *MiargB* and *MiglnB* ORFs lacking a stop codon. Wild-type tobacco leaves were used as negative controls. The fluorescence image of chloroplast autofluorescence is shown in red, whereas the fluorescence image of GFP is shown in green. The bright field image shows the light micrograph of the tobacco leaf epidermis, and the merged image overlays the above-described signals. The scale bar corresponds to 10 μm.

The subcellular localization of MiNAGK was estimated by transient expression in *Nicotiana benthamiana* leaves via agroinfiltration. The complete ORF of MiNAGK was ligated into the pCAMBIA1300-based expression binary vector pC1300-GFP to generate pC-NAGK-GFP (Supplementary Fig. S8). The resulting construct was infiltrated into the lower epidermal cells of tobacco leaves via *Agrobacterium tumefaciens* GV3101. The green fluorescence from GFP, as observed by using a confocal laser scanning microscope, was thoroughly dispersed in the epidermal cytoplasm including chloroplasts infected by the construct pC1300-GFP, which was used as a control (Fig. 5). By contrast, after *MiargB* was introduced, the green fluorescence signal was mainly visualized in the chloroplasts of infected cells, thus resulting in an overlap of the green fluorescence and the red autofluorescence of the chlorophylls (Fig. 5). So did the transformed plants infected by using expression binary vector carrying *MiglnB*, which is consistent with the immunoelectron microscopic examination (Figs 4 and 5). This result indicated that both NAGK and PII from *M*. *incisa* were located in the chloroplasts of infected tobacco leaf epidermal cells.

### MiNAGK-specific activity and Arg level changes in *M*. *incisa* during nitrogen starvation

**Figure 6:**
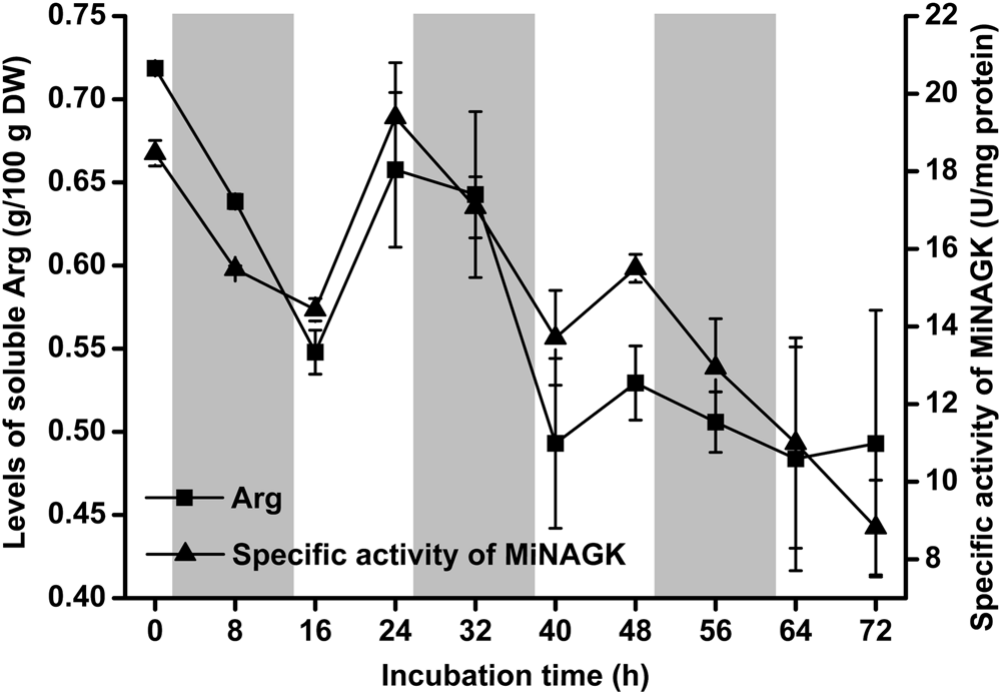
The levels of soluble Arg and the specific activity of MiNAGK in *Myrmecia incisa* cultured under nitrogen starvation stress. The region shadowed in gray denotes the night phase of a day. Each bar represents triplicate assessments that are presented as the means ± standard deviations (SD).

We examined the enzymatic activity of NAGK, which has been reported to catalyze the main control step in the Arg biosynthesis pathway^17,21,37^, in *M*. *incisa* during the shift culture from BG-11 medium to nitrogen starvation. MiNAGK began to decline from onset (e.g., 0 h) of the culture shift, but there was an unexpected increase at 24 h and 48 h (Fig. 6), at 19.390 U (mg protein)^−1^ and 15.499 U (mg protein)^−1^, respectively. At the end (e.g., 72 h) of this experiment (Fig. 6), the specific activity of MiNAGK decreased to the lowest level of 10.995 U (mg protein)^−1^.

During the process of nitrogen starvation, the changes in both soluble and hydrolyzed amino acid levels in *M*. *incisa* were examined with an emphasis on Glu and Arg, the initial reactant and terminal product in the Arg biosynthesis pathway. The total hydrolyzed amino acid levels appeared to be altered without any statistical significance, whereas the level of Arg showed a tendency towards a decrease (Supplementary Table S2). This decrease in Arg levels appeared to be more significant for the soluble amino acids in *M*. *incisa* (Fig. 6 and Supplementary Table S3). The shift to nitrogen starvation altered the soluble Arg levels along with a change in the enzymatic activity of MiNAGK (Fig. 6), both of which were found to be extremely significantly correlated (*r* = 0.854) at *P* = 0.002 < 0.01 by using SPSS. MiNAGK was thus thought to be responsible for the production of soluble and bound Arg because soluble and hydrolyzed Arg were also significantly positively correlated (*r* = 0.708, *P* = 0.022 < 0.05). In addition, the levels of Glu as a major soluble amino acid showed the same tendency as Arg.

## Discussion

In the present study, the glnB-type PII signal transduction protein in the arachidonic acid-rich green microalga *Myrmecia incisa* ^10^ was characterized. Amino acid sequence alignment (Supplementary Fig. S1) of MiPII and other homologues of the PII protein family showed their three characteristic T-, B-, and C-loops, as reviewed by Forchhammer and Lüddecke^38^. Like *Chlamydomonas reinhardtii* and *Arabidopsis thaliana*, MiPII also possesses conserved N- and C-terminal extensions (Supplementary Fig. S1), among which the latter contains a Q-loop, as described by Chellamuthu *et al*.^36^. MiPII is located in chloroplasts, as predicted by the presence of a chloroplast signal peptide (Supplementary Fig. S1) and verified by both immunoelectron microscopy (Fig. 4) with purified prepared anti-MiPII polyclonal antibody (Supplementary Fig. S7) and agroinfiltration of tobacco leaves (Fig. 5). Because of the large and irregular chloroplasts in *M*. *incisa*, we were unable to isolate chloroplasts and screen for PII-interacting proteins by using PII affinity chromatography. However, using database STRING (http://string-db.org) (data not shown), protein-protein interactions between MiPII and MiNAGK are proposed. This interaction was confirmed not only by *in vivo* yeast two-hybrid assay (Fig. 2F), but also by *in vitro* co-immunoprecipitation assay (Fig. 2G) and kinetics analysis of enzyme-catalyzed reactions (Fig. 3). However, using yeast two-hybrid assays, MiPII was found not to interact with MiBCCP, which to date has been identified only in *Arabidopsis thaliana* ^25^, *Synechocystis* sp. PCC 6803^26^, *Escherichia coli*, and *Azospirillum brasiliense* ^27,28^.

Two genes encoding BCCP subunits of *M*. *incisa* ACCase were cloned in the present study. MiBCCP1 has a characteristic biotin binding motif (194EAMKLMNEIE204) at its C-terminus (Supplementary Fig. S5), whereas MiBCCP2 lacks this motif with the conserved residue Lys for covalent binding of biotin, as reviewed by Cronan and Waldrop^29^. Because PII interacts with a biotinylated BCCP in *Arabidopsis thaliana* ^25^, it is reasonable to conclude that MiBCCP2 does not interact with MiPII, as detected by yeast two-hybrid analysis (Fig. 2D and E). Since the results obtained for *Arabidopsis thaliana* by Feria Bourrellier *et al*.^25^, the PII-BCCP interaction has been verified in only the bacteria *E*. *coli* and *Azospirillum brasiliense* ^27^ and cyanobacterium *Synechocystis* sp. PCC 6803^26^ by using protein co-precipitation; this interaction has not been reported in eukaryotic microalgae. This lack of information might reflect the difficulty associated with exploring the comprehensive PII-BCCP interaction. Ser49 in the T-loop of *Synechocystis* PII has been found to be a critical residue for PII-BCCP complex formation via phosphorylation/dephosphorylation^26^, although PII is not regulated by phosphorylation in *Arabidopsis* ^39^. The corresponding position of Ser49 is replaced by Thr in *M*. *incisa* as well as in *Chlamydomonas* PII proteins (Supplementary Fig. S1). Whether this change is responsible for the undetected MiPII-MiBCCP1 interaction in the present study remains unsolved. In addition, protein co-precipitation appears to be a better approach than the yeast two-hybrid assay used herein for clarification of the PII-BCCP interaction, because yeast has been reported to lack biotin ligase for biotinylation of BCCP^40^. Thus, the interaction of MiPII with MiBCCP1, which directly regulates fatty acid biosynthesis in *M*. *incisa*, requires further evidence.

In addition to the PII-BCCP interaction, it is well known that the interaction of PII protein with NAGK regulates Arg biosynthesis in cyanobacteria^14,21^ and higher plants^17–20^. This global C/N regulation system is completed phylogenetically by the *in vivo* and *in vitro* evidence obtained from the green microalga *M*. *incisa* (Figs 2 and 3) that is regarded as a mediator during the evolution from cyanobacteria to higher plants. MiNAGK has a PII signature motif between Lys104 and Gly151 (Supplementary Fig. S4). Furthermore, several corresponding residues (Supplementary Fig. S4) that participate in interaction of MiPII with MiNAGK are highly conserved, as revealed in comparison of the analyzed PII-NAGK complex crystal structure in *Synechococcus elongatus* strain PCC7942^41^ and *Arabidopsis thaliana* ^42^. For instance, the chains of Arg118 and Ser122 (corresponding to Arg126 and Thr130 in MiPII, as shown in Supplementary Fig. S1) in the T-loop of *Arabidopsis* PII form hydrogen bonds with the main chain carbonyl and amide groups of Val158 and Val161 (corresponding to Val171 and Val174 in MiNAGK, as shown in Supplementary Fig. S4) in *Arabidopsis* NAGK^42^. Apart from these residues, the NAGK residues Glu197, Arg236, Arg257, and Gln261, which are involved in the PII-NAGK interaction, and Ala260, which centers the hydrophobic patch linking both cyanobacterial proteins^41^, are highly conserved in MiNAGK, as shown in Supplementary Fig. S4. They correspond to the MiNAGK residues Glu263, Arg302, Arg323, Gln327, and Ala326, respectively (Supplementary Fig. S4). These conserved residues in MiPII and MiNAGK may enable these two proteins to bind to each other in this green microalga, thus allowing for their normal physiological actions in the algal cells.

MiPII interacts with MiNAGK and hence can function biologically only when both are located in the same compartment of a microalgal cell. Thus, MiPII and MiNAGK are predicted to be located in the chloroplasts of *M*. *incisa* because they both possess a chloroplast signal peptide at their N-termini (Supplementary Figs S1 and S4). Subsequently, MiPII was found to be located in chloroplasts by using both immunoelectron microscopy with the prepared anti-MiPII polyclonal antibody (Fig. 4) and agroinfiltration of tobacco leaves (Fig. 5). The results mirror that obtained for *Arabidopsis* and *Chlamydomonas* PII proteins^30,43^, as illustrated by Western blot analysis using proteins extracted from isolated chloroplasts. Similarly to *Arabidopsis* and rice NAGKs^17,18^, MiNAGK is also located in microalgal chloroplasts, as detected by agroinfiltration into tobacco leaves (Fig. 5). In comparison to previous findings^17,18^, no novel findings were obtained, but the present study is the first demonstration of the subcellular localization of NAGK in microalgae. In general, the identified subcellular localization of MiPII and MiNAGK spatially provides a possibility for the interaction of these two proteins to allow their normal functions.

**Figure 7:**
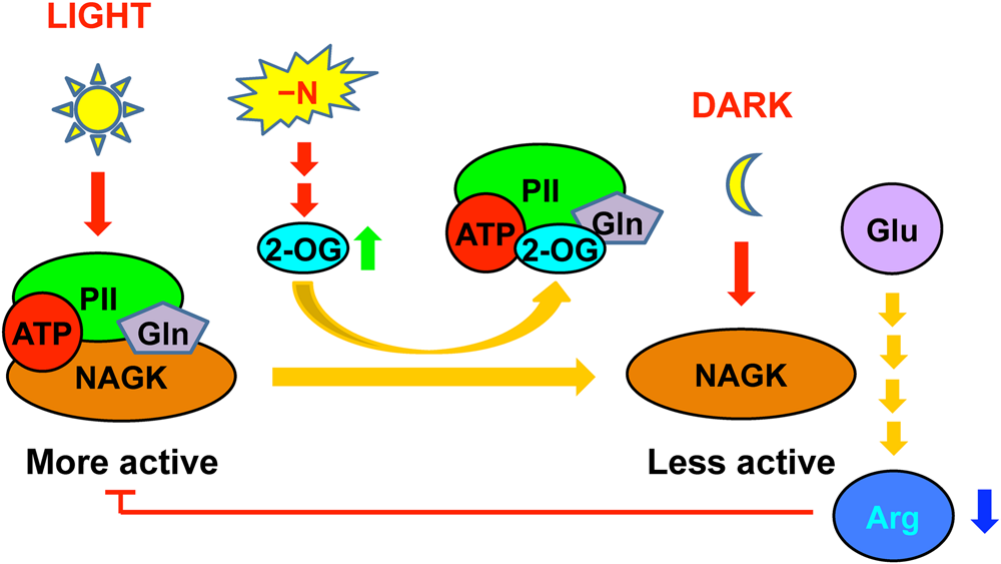
A proposed model showing the dissociation of MiPII from MiNAGK that results in decreased Arg levels in *Myrmecia incisa* in response to nitrogen starvation. Orange arrow denotes the biochemical reaction; Red one indicates the provision of signal; Green and blue ones denote the increase and decrease of products, respectively; and Red line indicates the inhibitory effect of Arg on MiPII-MiNAGK interaction.

Among the detected soluble amino acids in *M*. *incisa* (Supplementary Table S3), Arg is predominant in addition to Glu, thus confirming the suggestion that Arg is suitable as a storage form of organic nitrogen because it has the highest nitrogen-to-carbon ratio^44^. This storage form is reflected by comparisons between the levels of soluble and hydrolyzed Arg, the latter of which is exceeded by Glu, Asp, Leu, and even Val in hydrolyzed amino acids as protein components in this microalga (Supplementary Tables S2 and S3). Given that NAGK is a critical enzyme for catalyzing the biosynthesis of Arg, as reviewed by Llácer *et al*.^37^, and the extremely significantly positive correlation (*r* = 0.854, *P* = 0.002 < 0.01) between NAGK activity and soluble Arg levels in *M*. *incisa* during nitrogen starvation stress, NAGK is thought to be responsible for the regulation of Arg anabolism. Because MiNAGK can interact with MiPII in the chloroplasts of *M*. *incisa* as identified above, Arg biosynthesis is reasonably regulated by MiPII, which can sense the energy status and C/N ratio, as reviewed by Osanai and Tanaka^13^, Chellamuthu *et al*.^16^, and Forchhammer & Lüddecke^38^. Thus, the interaction of MiPII with MiNAGK can upregulate Arg biosynthesis in *M*. *incisa* by relieving the feedback inhibition of Arg when nitrogen is abundant (Fig. 3), as suggested by Ferrario-Méry *et al*.^19^ and Chellamuthu *et al*.^36^. This interaction, however, is antagonized by increased 2-OG (Figs 2 and 3), thus supporting the inhibitory effect of 2-OG on the activity of PII protein^45^. As a result, the Arg levels begin to decline when this microalga is cultured under nitrogen starvation stress (Fig. 6). Although the downward trend in Arg levels cannot be changed, Arg levels in *M*. *incisa* increase temporarily during exposure to light (Fig. 6), possibly because of both increased Mg^2+^ in the stroma imported from thylakoids^46^ and ATP generated by photophosphorylation under illumination^47^. The model for manipulation of Arg biosynthesis via MiPII in *M*. *incisa* chloroplasts in response to nitrogen starvation is proposed in Fig. 7, but the chloroplast rather than cellular NAGK activity and Arg levels appear to provide a better basis for the model establishment.

In addition to ATP, Mg^2+^, and 2-OG as indicated in Fig. 7, Gln can be sensed by PII as reported in *Chlamydomonas reinhardtii* and several higher plants including *Arabidopsis* ^36^ and, recently, *Chlorella variabilis* NC64A^48^. Glutamine binding to a C-terminal Q-loop in *Chlamydomonas* PII protein alters the conformation of PII, promoting the interaction and activation of NAGK^36^. Conservation of the C-terminal Q-loop in MiPII (Supplementary Fig. S1) implies that this PII protein could interact with Gln and subsequently promote the interaction with and activation of NAGK in *M*. *incisa*, which is confirmed by the present study (Fig. 3). This mechanism, however, appears to be less effective for the enhancement of MiNAGK activity because of the insignificant correlation between MiNAGK activity and the soluble Gln levels (*r* = 0.377, *P* = 0.283 > 0.05), as well as the very low levels of soluble Gln (approximately 0.4 to 1.7 μM estimated on the culture basis), which is approximately 10^3^ lower than the estimated half-maximal effective concentration (about 3 mM of Gln from Fig. 3, upper panels) in this microalga.

As reported by Ouyang *et al*.^10^, Chen *et al*.^33^, and Liu *et al*.^49^, *M*. *incisa* can accumulate arachidonic acid and TAG during culture under nitrogen starvation stress. The present findings indicated that, under this growth condition, the dissociation of MiPII from MiNAGK in microalgal chloroplasts leads to a decrease in the level of Arg. These findings raise questions concerning the relationship of these two physiological phenomena. A *Chlamydomonas* PII mutant generated by artificial microRNA has been reported to negatively control TAG accumulation in lipid bodies during acclimation to nitrogen starvation^32^. In a PII mutant strain of *Synechocystis* strain PCC 6803, Hauf *et al*.^26^ have found that the lack of control leads to lower acetyl-CoA levels and slightly higher levels of fatty acids and lipid body formation. These results imply a possible relationship between lipid and Arg biosynthesis via MiPII. If MiPII binds to MiBCCP, this complex could reasonably regulate the *de novo* biosynthesis of fatty acids in microalgal chloroplasts, but this interaction was not found in the present study. On the basis of the results reported for microalgal PII mutants^26,32^ and our findings, the ATP and 2-OG saved from Arg biosynthesis is proposed to facilitate the accumulation of fatty acids and TAG in *M*. *incisa* during exposure to nitrogen starvation. Obviously, a PII null mutant of *M*. *incisa* is expected to provide the direct evidence for exploration of the relationship between lipid and protein biosynthesis.

## Methods

### Algal strains and growth conditions

Strain H4301 of *Myrmecia incisa* Reisigl was obtained from the Culture Collection of Algae of Charles University of Prague. This microalga was cultivated in 800-mL glass flasks containing BG-11 medium^50^ at 25 °C under a 12-h light/12-h dark regime as described previously^34^. During culture, the flasks were shaken several times daily by hand at regular intervals.

For the nitrogen starvation studies, late logarithmic phase cultures (approximately 14 d) were centrifuged at 5,000 revolutions per min (rpm) for 10 min at room temperature, and the cell pellets were washed three times with fresh BG-11 medium without N. The pellets were then re-suspended in N-free medium in 500-mL cylindrical glass columns that were illuminated on one side with cool-white fluorescent Philips tubes (36 W) (Yizheng, Jiangsu, China) at a light irradiance of 115 μmol photons m^−2^·s^−1^ with aeration for another 72 h. The microalgal cells were sampled every 8 h by centrifugation and washing as described above for the detection of amino acids and NAGK specific activity.

### Gene cloning of *MiglnB*, *MiargB*, and *MiaccB*

From the transcriptome data of *M*. *incisa* ^34^, four contigs (Contig21987-10, Contig9491_6, Contig22797_3, and Contig10579_6) were searched, and they corresponded to the coding sequences of PII, NAGK, and BCCP. According to these sequences, primers (Supplementary Table S1) were designed by using Primer Premier 5.0 software (http://www.premierbiosoft.com/primerdesign/index.html) for cDNA cloning.

Total RNA was extracted from the collected microalgal cells using TRIzol reagent (Invitrogen, USA) according to the manufacturer’s protocol. The first-strand cDNA was synthesized by using the First-Strand Synthesis System for RT-PCR kit (TaKaRa, Dalian, China) according to the manufacturer’s guidelines. Both the 5′- and 3′-ends of the cDNA sequences of *MiglnB* were cloned using a SMART RACE cDNA Amplification Kit (Clontech, USA), according to the manufacturer’s instructions, with primers 5GSP1, 5GSP2, and 3GSP (Supplementary Table S1).

Full-length cDNA of *MiargB* was cloned from the above-synthesized first-strand cDNAs with the designed primers NAGK-F1 and NAGK-R1 (Supplementary Table S1). Both the 5′- and 3′-ends of the cDNA sequences of *MiaccB1* were cloned with primers BCCP1-5, BCCP1-3, and BCCP1-3N, whereas the *MiaccB2* cDNA was amplified with the primer pair BCCP2-F and BCCP2-R (Supplementary Table S1).

Genomic DNA was extracted from the freshly harvested microalgal cells by using the plant DNA kit (Tiangen, Beijng, China) according to the manufacturer’s protocol. One pair of primers, PII-F and PII-R (Supplementary Table S1), was designed as described above on the basis of the assembled cDNA of *MiglnB* and was used to clone the genomic DNA of *MiglnB*.

The PCR products were resolved on a 1.0% low-melting-point agarose gel for cDNA or DNA recovery. The target product was recovered using an agarose gel purification and extraction kit (Aidlab, China) and was ligated to a pMD19-T vector (TaKaRa, Dalian, China). The constructed vector was subsequently transformed into *Escherichia coli* JM109 competent cells (TaKaRa, Dalian, China) for blue-white selection. The positive clones were selected and sent to Sangon (Shanghai, China) for sequencing.

### Bioinformatics analysis of *MiglnB*, *MiargB*, and *MiaccB*

The detailed bioinformatics analysis is provided in the Supplementary Methods.

### Yeast two-hybrid assay

Strain AH109 of *Saccharomyces cerevisiae* and vectors pGBKT7, pGADT7, pGBKT7-53, pGADT7-T, and pGBKT7-Lam were commercially provided by Clontech Laboratories Inc. (CA, USA) for protein-protein interaction analysis. For the construction of bait and prey vectors, the oligonucleotide primers, as shown in Supplementary Table S1, were used for gene amplification of *MiglnB*, *MiargB*, *MiaccB1*, and *MiaccB2* lacking their signal peptide sequences. The amplicon carrying the mature MiPII-coding sequence was ligated into the *EcoR*I- and *Pst*I-digested pGBKT7 to generate the bait vector pGBKT7-glnB. Similarly, *MiargB*, *MiaccB1*, and *MiaccB2* lacking their signal peptide sequences were ligated into *EcoR*I- and *Xho*I-treated pGADT7 to generate the prey vectors, pGADT7-argB, pGADT7-accB1, and pGADT7-accB2, respectively. The plasmid pair between pGBKT7-glnB and pGADT7-argB, pGADT7-accB1, or pGADT7-accB2 was co-transformed into AH109 yeast separately by electroporation (Bio-Rad, USA), which were inoculated on SD-Trp-Leu plates according to the Clontech Yeast Protocols Handbook (Clontech, CA, USA). Expression of the *lac*Z reporter gene was determined by measuring β-galactosidase activity using 2-nitrophenyl-β-D-galactopyranoside as a substrate. The plasmid pairs pGBKT7-53 and pGADT7, and pGBKT7-Lam and pGADT7-T, served as a positive or negative control, respectively.

### Prokaryotic expression of MiPII and MiNAGK and preparation of MiPII polyclonal antibody

The cDNAs of the *MiglnB* and *MiargB* genes lacking signal peptide sequences were cloned by using two pairs of designed primers: ePII-F and ePII-R, and NAD-F and NAD-R (Supplementary Table S1), respectively. The amplified products were ligated into the *EcoR*I- and *Xho*I-digested His-tag fusion vector pET-28a to generate the recombinant vectors pET-PII or pET-NAGK. Overexpression of the recombinant protein in transformed *E*. *coli* BL21 was induced with 0.1 mM isopropyl-β-D-thiogalactoside (IPTG) at 30 °C for 4 h. Pelleted cells were dissolved in buffer (50 mM Tris-HCl, pH 8.0, 50 mM NaCl, 1 mM EDTA, 10 mg/mL lysozyme). Phenylmethanesulfonyl fluoride (PMSF, 1 mM) was added before cell lysis to protect the target products from degradation by protease. The soluble fraction was subjected to metal-chelate affinity chromatography using Ni-NTA resin (Qiagen, Hilden, Germany) according to the manufacturer’s instructions. Denaturing SDS-PAGE was performed, and the protein was quantified using the Bradford method^51^.

Antisera were obtained from rabbits that had been immunized with the purified recombinant protein MiPII from *E*. *coli* transformed with pET-PII. The MiPII polyclonal antibody was purified according to the method described by Olmsted^52^ and Ritter^53^.

### Western blot analysis

Western blot procedures were carried out essentially as described previously^54^. The total proteins extracted from microalga or transformed *E*. *coli* were separated by 12% SDS-PAGE. They were then electronically transferred from the gel to a nitrocellulose membrane. The protein blot was blocked in 5% skim milk powder in Tris-buffered saline solution with 0.1% Tween 20 (TBST, 0.025 M Tris, 0.137 M NaCl, 2.7 mM KCl, and 0.1% Tween 20 at pH 7.4) overnight at 4 °C. After being washed, the membrane was incubated with purchased anti-His antibody (Tiangen Biotech Co., Ltd., Bejing) or purified anti-MiPII polyclonal antibody diluted 1:3,000 in TBST for 1 h at room temperature, and this was followed by a 1:3,000 dilution in TBST of anti-rabbit IgG secondary antibody labeled with horseradish peroxidase (Youke Biotechnology Co., Ltd., Shanghai). The color reaction was visualized with a diaminobenzidine kit (Tiangen Biotech Co., Ltd., Bejing) according to the manufacturer’s instructions.

### Co-immunoprecipitation assay

Co-immunoprecipitation was carried out using protein A Sepharose (Amersham Pharmacia Biotech, UK) to detect the *in vitro* interaction between MiPII and MiNAGK. Protein A Sepharose was washed three times with 5 volumes of TBST. To 1 mL of the solution (50 mM Tris-HCl, pH 7.5, 100 mM KCl) were added 200 μg and/or 400 μg of the recombinant MiPII and/or MiNAGK proteins, respectively, in the presence or absence of ATP (3 mM), Mg^2+^ (6 mM), and 2-OG (5 mM). The supernatant was incubated (with constant shaking) with rabbit anti-MiPII polyclonal antibody at room temperature for 1 h, and this was followed by the addition of SPA (Roche) and incubation at 4 °C for another 1 h according to the manufacturer’s instructions. Pelleted beads were resuspended in 10 × SDS loading buffer, boiled, and subjected to 15% SDS-PAGE. Proteins in the gel were detected by Coomassie blue R-250 staining, and the intensity of target band was estimated by using ImageJ software (http://imagej.nih.gov/ij/).

### Subcellular localization of MiPII and MiNAGK

The detailed subcellular localization of MiPII and MiNAGK is provided in the Supplementary Methods.

### Detection of MiNAGK activity and amino acids

NAGK activity in *M*. *incisa* cultured under nitrogen starvation was determined as described by Dénes^55^ with slight modifications. The crude enzyme from the freshly harvested *M*. *incisa* was extracted with a solution composed of 50 mM Tris-HCl (pH 7.4), 4 mM EDTA, 1 mM dithiothreitol, and 0.5 mM benzamidine. The reaction was started by mixing an equal volume (200 μL) of 80 mM *N*-acetyl-L-glutamate, the crude enzyme, and a reaction solution containing 0.8 M NH_2_OH-HCl, 40 mM MgCl_2_, and 20 mM ATP. After incubation at 37 °C for 1 h, the reaction was stopped by the addition of 400 μL of a solution containing 5% FeCl_3_·6H_2_O, 8% trichloroacetic acid, and 0.3 M HCl. Absorbance of the generated hydroxamate·Fe^3+^ complex in the reaction was measured at 540 nm with a NanoDrop 2000C photospectrometer (Thermo, USA) after the removal of precipitated proteins by centrifugation. The molar extinction coefficient of this complex was 456 M^−1^ cm^−1^, as reported by Haas and Leisinger^56^ for the calculation of MiNAGK activity. One unit refers to the amount of enzyme required to catalyze the formation of 1.0 micromole of *N*-acetylglutamate 5-hydroxamate in 1 h at 37 °C^55^, and the specific activity of MiNAGK is expressed as units per milligram of protein. The data shown are the results of at least duplicate assays.

The activity of the recombinant MiNAGK and the effects of Arg (from 0 to 6 mM), 2-OG (from 0 to 4 mM), or Gln (from 0 to 12 mM) on this activity were studied similarly to this detection, but the 0.6 mL of reaction solution contained 0.5 mM dithiothreitol and 5 μg MiNAGK instead of crude enzyme. In addition, this reaction solution included 10 μg MiPII and 10 mM Gln when they were needed.

Lyophilized microalgal material (5 mg DW) was used for metabolite extraction in a three-step ethanol-water procedure using successively 500 μL of 80% (v/v) ethanol, 500 μL of 60% (v/v) ethanol and 500 μL water at 4 °C for 60 min. The supernatants of the different extraction steps were collected after centrifugation at 12,000 × g and mixed thoroughly^57^. Quantification of the main soluble amino acids (Arg, Asn Asp, Gln, Glu, and His) in the supernatants was performed using Agilent 1200 Series (USA) ion exchange chromatography. After the extraction of soluble amino acids, the pellets were lyophilized and hydrolyzed with 6 M HCl at 110 °C for 22 h. After filtering, the bound amino acids were quantified by using a Bichrom 30 amino acid analyzer (Hitachi, UK). The contents of amino acids (g/100 g DW) are expressed as the means ± standard deviations of triplicate assessments.

## Electronic supplementary material

Supplementary Information
