# Supplementary material for: Evidence for PII with NAGK interaction that regulates Arg synthesis in the microalga Myrmecia incisa in response to nitrogen starvation
Source: Sci Rep. 2017 Nov 24;7:16291. doi: 10.1038/s41598-017-16644-3 (PMC5701185; doi:10.1038/s41598-017-16644-3)
Supplement: Supplementary file 1 — Supplementary Information [file 41598_2017_16644_MOESM1_ESM.pdf]

Supplementary Information

Evidence for PII with NAGK interaction that regulates Arg synthesis in the microalga *Myrmecia incisa* in response to nitrogen starvation

Yan Li[1], Wei Liu[1], Li-Ping Sun[1], Zhi-Gang Zhou[1, 2, 3]*

[1]Key Laboratory of Exploration and Utilization of Aquatic Genetic Resources Conferred by Ministry of Education, Shanghai Ocean University, Shanghai 201306, China

[2]National Demonstration Center for the Experimental Teaching of Fisheries Science, Shanghai Ocean University, Shanghai 201306, China

[3]International Research Center for Marine Biosciences Conferred by Ministry of Science and Technology, Shanghai Ocean University, Shanghai 201306, China

*Author for correspondence: e-mail zgzhou@shou.edu.cn. Tel: 0086-21-61900424, Fax: 0086-21-61900405

Supplementary Methods

Bioinformatics analysis of *MiglnB, MiargB,* and *MiaccB*

ORF Finder (http://www.ncbi.nlm.nih.gov/gorf/gorf.html) was used to predict the open reading frame (ORF) of the three genes *MiglnB*, *MiargB*, and *MiaccB*. The intron and extron of *MiglnB* were analyzed using Spidey (http://www.ncbi.nlm.nih.gov/spidey/). The amino acid compositions of their corresponding deduced proteins, MiPII, MiNAGK, and MiBCCP, were analyzed using BioEdit V 7.0.9.0 program[1]. Signal peptide site of MiPII, MiNAGK, and MiBCCP was predicted by both SignalP 4.1 Server (http://www.cbs.dtu.dk/services/SignalP/) and ChloroP 1.1 Server (http://www.cbs.dtu.dk/services/ChloroP/), while their subcellular location was predicted by TargetP 1.1 Server (http://www.cbs.dtu.dk/services/TargetP/) and PredAlgo webserver (http://giavap-genomes.ibpc.fr/predalgo). TMHMM Server v. 2.0 (http://www.cbs.dtu.dk/services/TMHMM/) was used to predict transmembrane domains. Protein sequences of PII, NAGK, and BCCP were retrieved from NCBI (http://www.ncbi.nlm.nih.gov/protein/?) separately, and the phylogenetic inference was constructed using MEGA 6.0 program with the neighbor-joining method[2]. Homologous sequences of PII, NAGK, and BCCP from different organisms were aligned independently by Clustal_X with default parameters[3].

Subcellular localization of MiPII and MiNAGK

Immunoelectron microscopy was employed to examine the precise subcellular localization of MiPII in *M. incisa* using the purified MiPII polyclonal antibody. The freshly collected microalgal cells were subject sequentially to pre-fixation (more than 2 h), post-fixation (3 h) in 2.5% glutaraldehyde, and 1% osmium tetroxide, respectively, in 0.1 M phosphate buffer at pH 7.4[4]. After dehydration in ethanol/acetone series, the fixed samples were then placed in acetone/Spurr’s resin (2/1, v/v) overnight, and then incubated in 100% Spurr’s resin for 2-3 h at 37°C. The embedded samples were polymerized in a temperature gradient and then thin-sectioned using a LKB 4802 ultrotome ultramicrotome (Leica, Germany).

The ultrathin sections used for immunoelectron microscopy were collected on 200-mesh nickel grids. The grids were washed for 2 min with water and transferred onto drops of balance buffer (BB, containing 50 mM PBS, 1% bovine serum albumin, 0.1 M NaCl, and 0.02% polyethylene glycol 20000) for 30 min at room temperature. Then they were incubated with the purified anti-PII antibody (diluted 1:1000 in BB) at 4°C for 48 h. After incubation, the grids were placed onto drops (repeated 3) of water for 3 min and another 30 min incubation with BB. Then the grids were incubated with the secondary antibody, anti-rabbit IgG conjugated to 10 nm gold particles (Sigma, USA) diluted 1:100 with BB at room temperature for 2 h. Following sequential washes in water and dehydration in the air, the sections were stained with 3% uranyl acetate-lead citrate, and observed in a JEOL-1230 Transmission Electron Microscope at 80 kV.

The labeling density was defined as the number of gold particles per area unit (μm^2) as described by Bernal[5], and the area was estimated by Adobe Photoshop software (ver. 3.0). Following counting the gold particles on chloroplasts and the other area calculated by subtracting the total area of chloroplasts in each micrograph, the percentage of particles versus the total number of particles was calculated. The statistical analysis of subcellular distribution of MiPII protein was carried out by using the independent sample test in SPSS Statistics 17.0 program.

Agroinfiltration of tobacco leaves was used as described by Liu *et al.*[6] for the determination of subcellular localization of MiNAGK and MiPII. The *MiargB* ORF was amplified from *M. incisa* using the pair of primers, NAD-F and NAD-R (Table S1), which

contains *Kpn*I/*Xba*I digestion sites. Similarly, the *MiglnB* ORF was amplified using the pair of primers, PIIGFP-F and PIIGFP-R (Table S1). The PCR product was digested with *Kpn*I and *Xba*I and subsequently introduced into the *Kpn*I/*Xba*I-digested pCAMBIA1300-3×GFP (from Prof. Z.-N. Yang) between the CaMV35S promoter and green fluorescent protein (GFP) gene to generate the corresponding fusion vector pC-NAGK-GFP or pC-PII-GFP. This recombinant vector and an empty plasmid, pC1300-GFP, as the negative control, were separately introduced into *Agrobacterium tumefaciens* GV3101 by electroporation (Bio-Rad, USA) and then infiltrated into the abaxial side of leaves of 4- to 5-week-old *Nicotiana benthamiana* plants. After agroinfiltration, the transgenic plant was placed in the dark for 48 h. The epidermis cells of the infiltrated tobacco leaves were observed under a confocal laser scanning microscope (Carl Zeiss or Leica, Germany). GFP fluorescence was monitored with a 500-nm to 550-nm band pass emission filter excited at 488 nm, and the autofluorescence of chlorophyll was examined with the same excitation filter as GFP, but with a 650-nm to 750-nm emission filter.

Supplementary References

1. Hall, T. A. BioEdit: a user-friendly biological sequence alignment editor and analysis program for Windows 95/98/NT. *Nucleic Acids Symp. Ser.* **41**, 95-98 (1999).
2. Tamura, K. *et al.* MEGA6: Molecular evolutionary genetics analysis version 6.0. *Mol. Biol. Evol.* **30**, 2725-2729 (2013).
3. Thompson, J. D. *et al.* The CLUSTAL_X windows interface: flexible strategies for multiple sequence alignment aided by quality analysis tools. *Nucl. Acids Res.* **25**, 4876-4882 (1997).
4. Ouyang, L.-L. *et al.* Expressed sequence tags analysis revealing the taxonomic position and fatty acid synthesis in an oleaginous green microalga, *Myrmecia incisa* Reisigl (Trebouxiophyceae, Chlorophyta). *Chin. Sci. Bull.* **57**, 3342-3352 (2012).
5. Bernal, M. *et al.* Identification and subcellular localization of the soybean copper P1B-ATPase Gm HMA8 transporter. *J. Struct. Biol.* **158**, 46-58 (2007).
6. Liu, X.-Y., Ouyang, L.-L. & Zhou, Z.-G. Phospholipid: diacylglycerol acyltransferase contributes to the conversion of membrane lipids into triacylglycerol in *Myrmecia incisa* during the nitrogen starvation stress. *Sci. Rep.* **6**, 26610 (2016).

Supplementary Table S1

Table S1. Nucleotide sequence of primers employed in the present study.

Primer	Nucleotide sequence from 5’ to 3’	Annealing temperature	Objective
cDNA cloning			
5GSP1	AATGCCGGGCTCCTGTTTCATA	68°C	*MiglnB*
5GSP2	CGTATCGCTCACCCTTTCCTCCCT	68°C	
3GSP	CAATGGTATCCTGGGCATGACCGTGAC	68°C	
NAGK-F1	CATATGCAGGCCAACACTCGT	59°C	*MiargB*
NAGK-R1	CTAGCCSGTGATCATGGTRCC		
BCCP1-5	GTCAACTCCTCAAAGTCCAACGGGT	64°C	*MiaccB1*
BCCP1-3	CGGGAGAGGGCAACGGTGTAC	64°C	
BCCP1-3N	CCTCCGCAGCAGTATGCACAAGCCC	63.7°C	
BCCP2-F	ATGGTCGCTTTGCAAGCG	60°C	*MiaccB2*
BCCP2-R	GCGTACTTGCTRTCSCCGAT		
DNA cloning			
PII-F	ATGGAGGTTTTCCAACTGCTG	59°C	*MiglnB*
PII-R	CTAAATGCCGGGCTCCTGTTT		
Heterologous expression in *Escherichia coli*			
ePII-F	GGAATTCGCCTGTGCCGGCAATGGG	57°C	*MiglnB* ORF minus transit peptide
ePII-R	CCGCTCGAGCTAAATGCCGGGCTCCTG		
NAD-F	CGGAATTCCAAGCGAGCGGTGCAGTT	66.5°C	*MiargB* ORF minus transit peptide
NAD-R	CCGCTCGAGCTAGCCGGTGATCATGGT		
Yeast two-hybrid analysis			
PIIBK-F	GGAATTCGCCTGTGCCGGCAATGGG	58°C	*MiglnB* ORF minus transit peptide
PIIBK-R	AACTGCAGCTAAATGCCGGGCTCCTG		
NAD-F	CGGAATTCCAAGCGAGCGGTGCAGTT	66.5°C	*MiargB* ORF minus transit peptide
NAD-R	CCGCTCGAGCTAGCCGGTGATCATGGT		
B1AD-F	CGGAATTCGCTCAGGAAGCCGATTCC	65.5°C	*MiaccB1* ORF minus transit peptide
B1AD-R	CCGCTCGAGTCAGGGCTTGATGATCAT		
B2AD-F	CGGAATTCGCAGCCCTGGTCACCCAG	65.5°C	*MiaccB2* ORF minus transit peptide
B2AD-R	CCGCTCGAGTGCATACTTGCTGTCGCC		
Subcellular localization by agroinfiltration of tobacco leaves			
NGFP-F	GGGGTACCATGCAGGCCAACACTCGT	61°C	MiNAGK
NGFP-R	GCTCTAGAGCCGGTGATCATGGTGCC		
PIIGFP-F	GGGGTACCATGGAGGTTTTCCAACTG	61°C	MiPII
PIIGFP-R	TGCTCTAGACTAAATGCCGGGCTCCTG		

The underlined letters in the primers denote the position of restriction enzyme sites.

Supplementary Table S2

Table S2. Levels of hydrolyzed amino acids (g/100 g dry weight) in *Myrmecia incisa* during the culture under nitrogen starvation. Shadowed data denote the minor one in the hydrolyzed amino acids.

Amino acid	0 h	8 h	16 h	24 h	32 h	40 h	48 h	56 h	64 h	72 h
Ala	3.074±0.0233	3.122±0.0634	3.120±0.0665	3.162±0.0520	3.224±0.0668	3.094±0.0520	3.088±0.0472	3.151±0.0266	3.077±0.1174	3.156±0.0712
Arg	2.944±0.0396	3.037±0.00451	3.034±0.0562	3.032±0.1325	3.040±0.1330	2.893±0.1205	2.919±0.0831	2.865±0.1080	2.760±0.1592	2.811±0.1719
Asp	3.829±0.0516	3.975±0.0600	3.925±0.0935	4.005±0.0835	4.053±0.0717	3.917±0.0223	3.966±0.0783	4.021±0.0313	3.935±0.1096	3.974±0.1391
Glu	3.934±0.0644	3.895±0.2725	4.000±0.0506	4.037±0.0917	4.211±0.1624	3.908±0.1125	4.173±0.1365	4.274±0.1258	4.058±0.0647	4.125±0.1160
Gly	2.354±0.0070	2.404±0.0473	2.391±0.0529	2.422±0.0451	2.483±0.0609	2.397±0.0367	2.370±0.0441	2.421±0.0200	2.353±0.1059	2.413±0.0576
His	0.803±0.0191	0.816±0.0248	0.815±0.0298	0.835±0.0035	0.859±0.0320	0.840±0.0236	0.804±0.0121	0.829±0.0326	0.801±0.0797	0.815±0.0100
Ile	1.765±0.0211	1.807±0.0626	1.833±0.0718	1.859±0.0184	1.950±0.0609	1.899±0.0272	1.710±0.0050	1.755±0.0458	1.745±0.0598	1.770±0.0685
Leu	3.831±0.0474	3.912±0.0812	3.898±0.0724	3.962±0.0712	4.089±0.0725	3.947±0.0115	3.869±0.1101	3.915±0.0341	3.905±0.1178	3.893±0.1203
Lys	2.547±0.0186	2.585±0.0578	2.599±0.0364	2.630±0.00751	2.673±0.0690	2.576±0.0225	2.568±0.0527	2.605±0.0430	2.586±0.173	2.634±0.0979
Met	0.369±0.0811	0.430±0.247	0.313±0.0678	0.228±0.0827	0.413±0.277	0.424±0.369	0.425±0.272	0.426±0.0841	0.405±0.230	0.322±0.182
Phe	2.697±0.0250	2.698±0.0540	2.668±0.00889	2.679±0.0447	2.730±0.101	2.653±0.0437	2.624±0.0693	2.699±0.0552	2.594±0.0828	2.647±0.144
Pro	2.123±0.0501	2.099±0.106	2.096±0.0750	2.111±0.0849	2.232±0.197	2.134±0.0799	2.245±0.123	2.240±0.104	2.223±0.0913	2.229±0.0710
Ser	1.921±0.0255	1.943±0.0427	1.903±0.0106	1.929±0.0613	1.920±0.0457	1.874±0.0356	1.971±0.0486	2.042±0.0140	1.990±0.0875	2.042±0.0361
Thr	2.159±0.0499	2.196±0.0344	2.198±0.0326	2.223±0.0490	2.236±0.0580	2.165±0.0378	2.198±0.0321	2.254±0.0197	2.202±0.0834	2.252±0.0510
Tyr	1.569±0.0331	1.580±0.0505	1.525±0.0118	1.535±0.0314	1.581±0.0449	1.534±0.00851	1.528±0.0318	1.576±0.0115	1.498±0.0373	1.536±0.0477
Val	3.188±0.120	3.259±0.0816	3.185±0.119	3.199±0.0366	3.208±0.0715	3.254±0.101	2.768±0.0640	2.845±0.188	2.833±0.255	3.004±0.123
Total	39.107±0.0327	39.760±0.6456	39.503±0.5748	39.848±0.6735	40.902±1.3277	39.509±0.6196	39.228±0.4640	39.918±0.4418	38.966±1.3884	39.621±1.3796

Supplementary Table S3

Table S3. Levels of several soluble amino acids (g/100 g dry weight) in *Myrmecia incisa* during the culture under nitrogen starvation. Shadowed data denote the major one in the detected soluble amino acids.

Amino acid	0 h	8 h	16 h	24 h	32 h	40 h	48 h	54 h	64 h	72 h
Ala	0.395±0.0154	0.207±0.00324	0.149±0.00334	0.162±0.0184	0.171±0.0332	0.121±0.00908	0.127±0.0101	0.120±0.0127	0.133±0.00904	0.137±0.00909
Arg	0.719±0.00226	0.638±0.00439	0.548±0.0133	0.658±0.0465	0.643±0.0499	0.493±0.0511	0.529±0.0223	0.506±0.0182	0.484±0.0673	0.493±0.0802
Asn	0.251±0.0112	0.242±0.00499	0.192±0.00669	0.212±0.0109	0.178±0.0181	0.125±0.0140	0.148±0.00186	0.119±0.00419	0.118±0.0177	0.126±0.0250
Asp	0.0333±0.0217	0.0142±0.00139	0.00656±0.000829	0.0311±0.00174	0.00245±0.00173	–*	0.0210±0.000112	0.00208±0.00147	0.00545±0.00358	0.0179±0.00620
Gln	0.0177±0.0125	0.0420±0.000447	0.0243±0.000358	0.0210±0.000442	0.0278±0.00268	0.0145±0.00294	0.0138±0.00109	0.0165±0.00139	0.0125±0.00301	0.0140±0.00167
Glu	0.806±0.0208	0.870±0.0192	0.813±0.0124	0.778±0.0119	0.716±0.00240	0.654±0.0677	0.776±0.0317	0.631±0.107	0.708±0.0325	0.685±0.0341
Gly	0.0520±0.00111	0.0619±0.000577	0.0309±0.000714	0.0338±0.00225	0.0587±0.00101	0.0306±0.000714	0.0309±0.00130	0.0310±0.000896	0.0584±0.00341	0.0571±0.00194
His	0.131±0.0229	0.298±0.0113	0.253±0.00659	0.286±0.0402	0.270±0.0378	0.200±0.0214	0.146±0.0183	0.116±0.00453	0.0959±0.0180	0.0677±0.0232
Lys	0.109±0.0100	0.153±0.00805	0.0365±0.00478	0.0244±0.0117	0.153±0.0162	0.0497±0.0118	0.0498±0.0217	0.0749±0.00847	0.123±0.0163	0.0760±0.0194
Ser	0.0488±0.00633	0.0689±0.00687	0.0579±0.00256	0.0675±0.00512	0.0653±0.0130	0.0478±0.00675	0.0412±0.00343	0.0421±0.00229	0.0377±0.00368	0.0104±0.0181
Val	0.0116±0.00292	0.0396±0.00379	0.00515±0.000707	0.0151±0.00605	0.0363±0.00973	0.0117±0.00662	0.00709±0.00711	0.00668±0.00279	0.0233±0.0119	0.00809±0.00737

* "–" means Undetectable.

Supplementary Figure S1

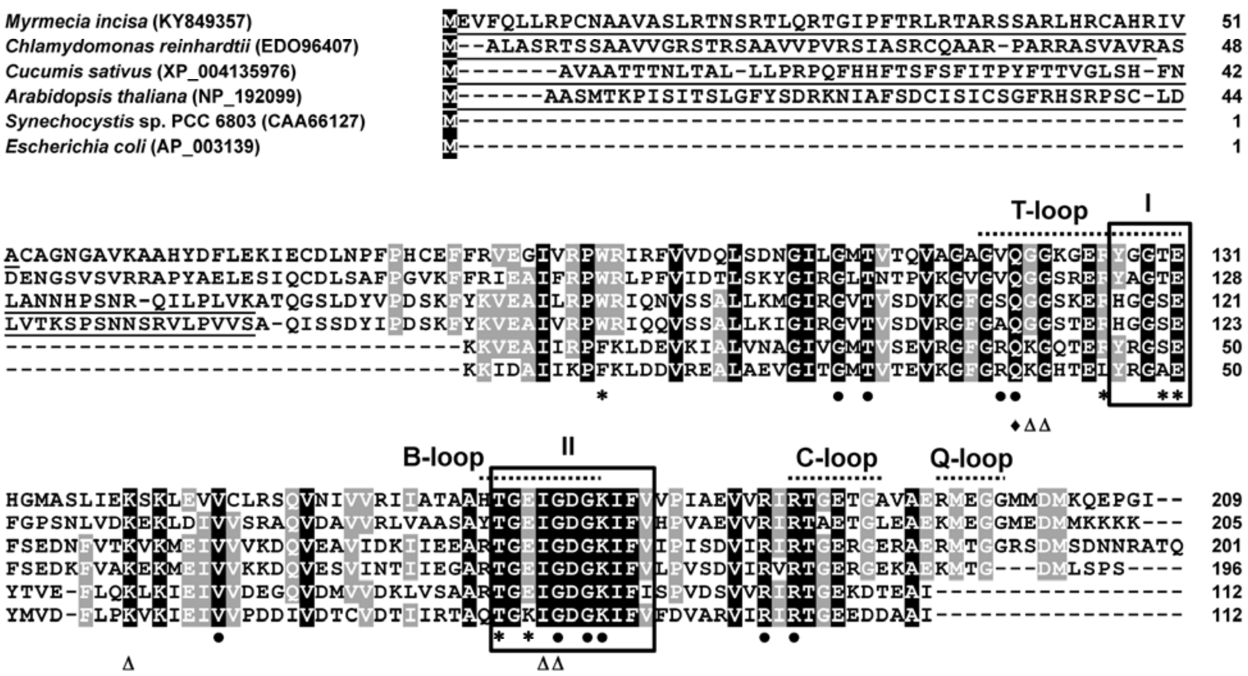

Figure S1. Alignment of the deduced amino acid sequences of PII proteins from *Myrmecia incisa* and other organisms.

Residues highlighted in black or gray backgrounds are identical or conserved in at least 60%, respectively, of all aligned PII proteins. The putative plastid-target sequence is indicated by underlining. Boxes I and II refer to PII signature patterns I and II. The position of the ATPase is indicated by black rhombus. ATP-, NAGK-, 2-OG-binding residues are denoted by black dot, asterisk and empty triangle, respectively.

Supplementary Figure S2

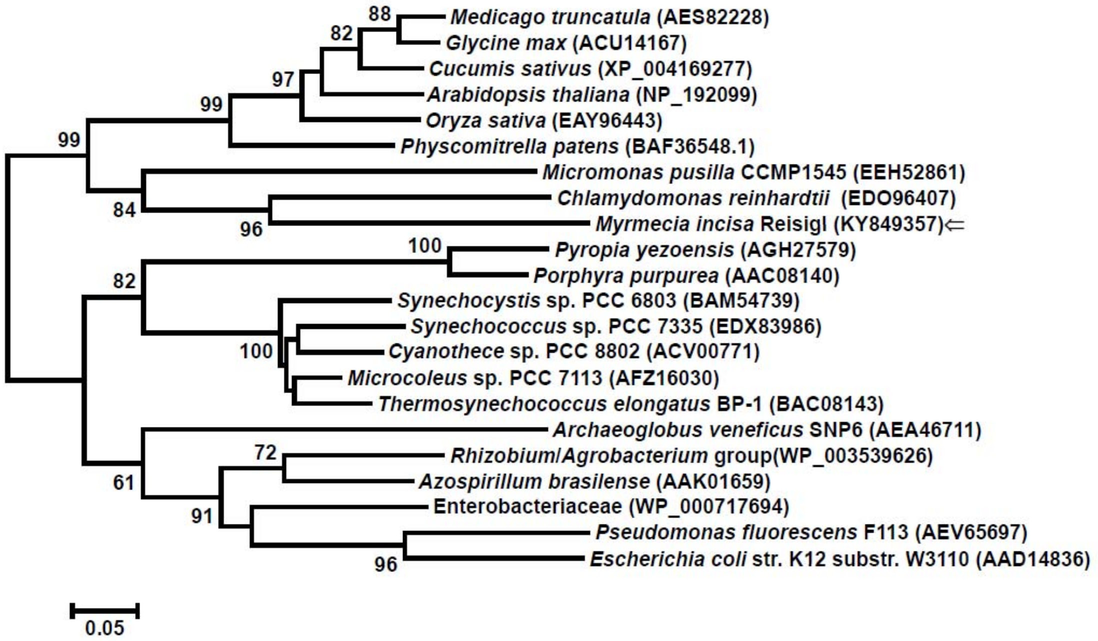

Figure S2. Neighbor-joining phylogenetic tree inferred from the amino acid sequences deduced from the nucleotide sequences of GlnB-type PII genes from *Myrmecia incisa* and other selected organisms.

All of the accession numbers are presented in the parentheses after the Latin names of each species. Branch lengths are proportional to the number of substitutions per site (see the scale bar). The numbers at the nodes indicate the neighbour-joining bootstrap proportion (BP) values (only values ≥50% are shown). The position of MiPII is marked by an arrow.

Supplementary Figure S3

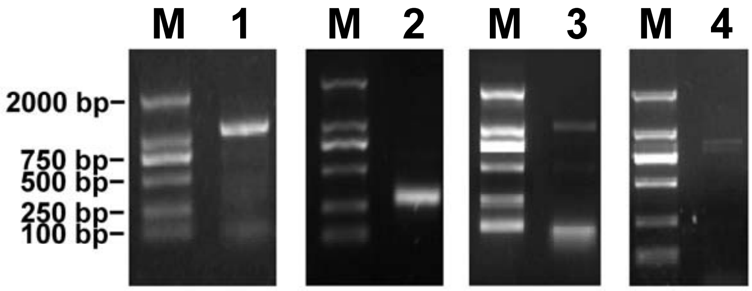

Figure S3. Electrophoresis patterns of the *MiargB* and *MiaccB* cDNA amplicons. Lane 1, cDNA amplicon of *MiargB*; Lane 2: 5’-RACE product of *MiaccB1*; Lane 3, 3’-RACE product of *MiaccB1*; Lane 4, product of *MiaccB2* ORF; and Lane M, DL 2000 DNA standard marker (Tiangen Biotech Co., Ltd.).

Supplementary Figure S4

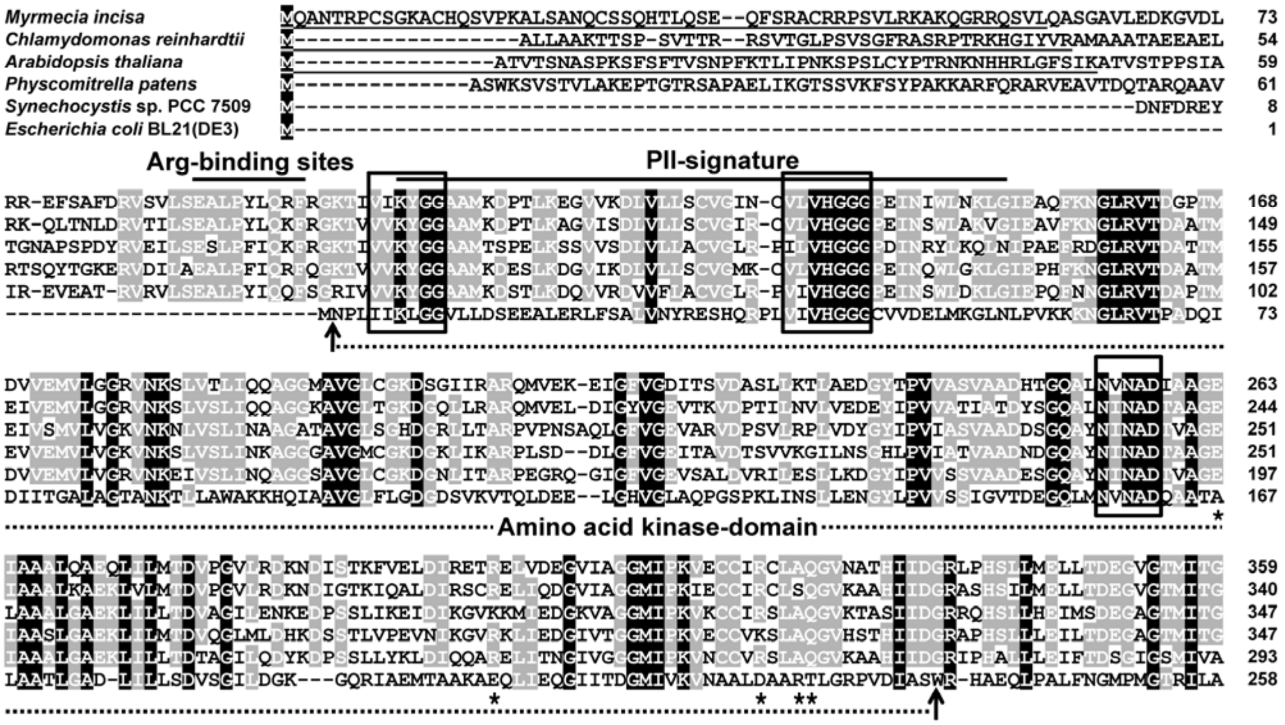

Figure S4. Amino acid sequence alignment of NAGK proteins from *Myrmecia incisa* and other organisms.

The accession numbers for *Chlamydomonas reinhardtii*, *Arabidopsis thaliana*, *Physcomitrella patens* subsp. *patens*, *Synechocystis* sp. PCC 7509, and *Escherichia coli* BL21(DE3) NAGKs used are EDP09199, AEE79672, EDQ63078, ELR86484, and 1GS5_A, respectively. Residues highlighted in black or gray backgrounds are identical or conserved in at least 60%, respectively, of all aligned NAGK proteins. PII binding residues are denoted by asterisks. The putative plastid-target sequence and the amino acid-kinase domain are indicated by the solid and dotted underlines, respectively. The conserved signature sequence of nitrogen regulatory protein PII and arginine-binding sites are indicated by overlines. The conserved sequences of the substrate-binding sites of NAGK are shown in boxes.

Supplementary Figure S5

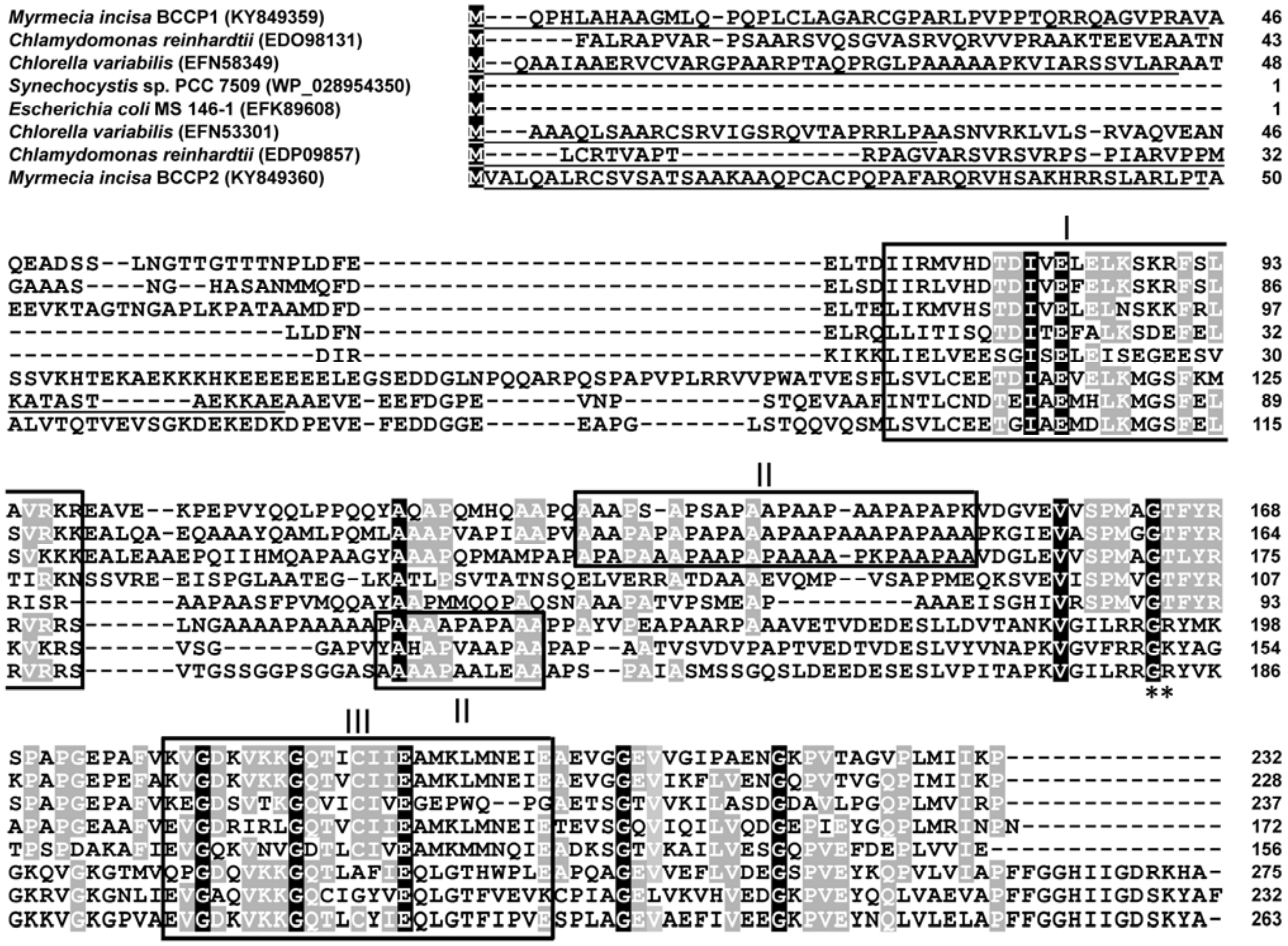

Figure S5. Multisequence alignment of the deduced BCCP proteins from *Myrmecia incisa* and other selected organisms.

Residues highlighted in black or gray backgrounds are identical or conserved in at least 60%, respectively, of all aligned BCCP proteins. The putative plastid-target sequence is indicated by underlining. Box denotes the conservative domain. The putative binding sites with biotin carboxylase subunit are denoted by asterisks.

Supplementary Figure S6

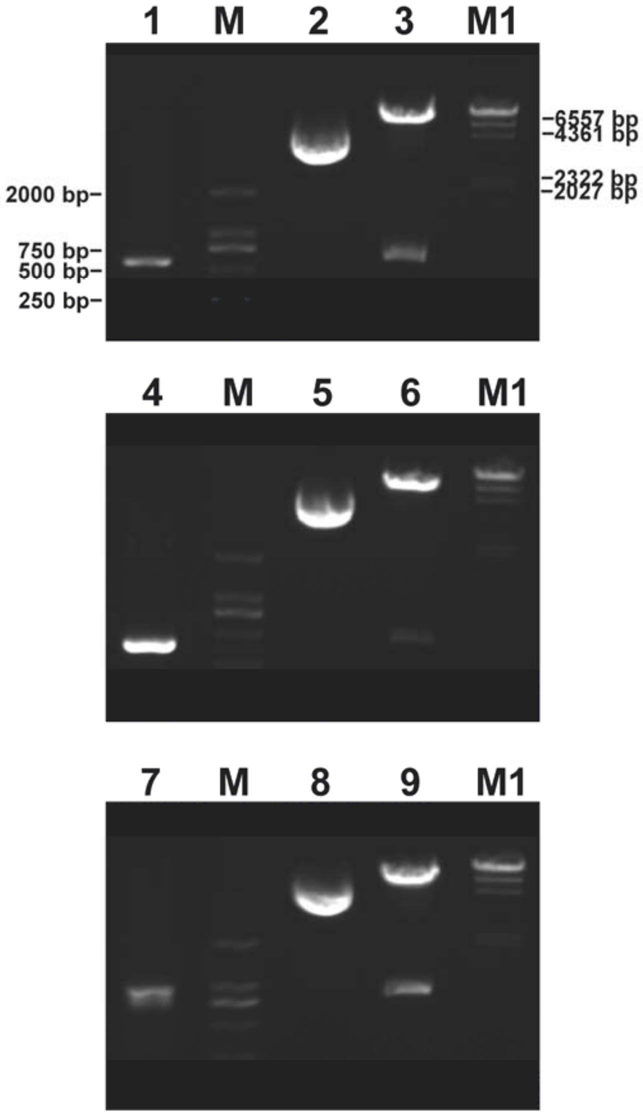

Figure S6. Electrophoresis patterns of the prey plasmids pGADT7-argB, pGADT7-accB1 and pGADT7-accB2 constructed for yeast two-hybrid analysis. Lanes 1, 4 and 7, amplified products of *MiargB*, *MiaccB1* and *MiaccB2*, respectively, ORFs minus signal peptide sequences; Lanes 2, 5 and 8, recombinant plasmids pGADT7-argB, pGADT7-accB1 and pGADT7-accB2, respectively; Lanes 3, 6 and 9, double digested products of plasmids pGADT7-argB, pGADT7-accB1 and pGADT7-accB2, respectively, with *Eco*RI and *Xho*I; Lane M, DL 2000 DNA standard marker (Tiangen Biotech Co., Ltd.); and Lane M1, λDNA *Hin*dIII DNA marker (Tiangen Biotech Co., Ltd.).

Supplementary Figure S7

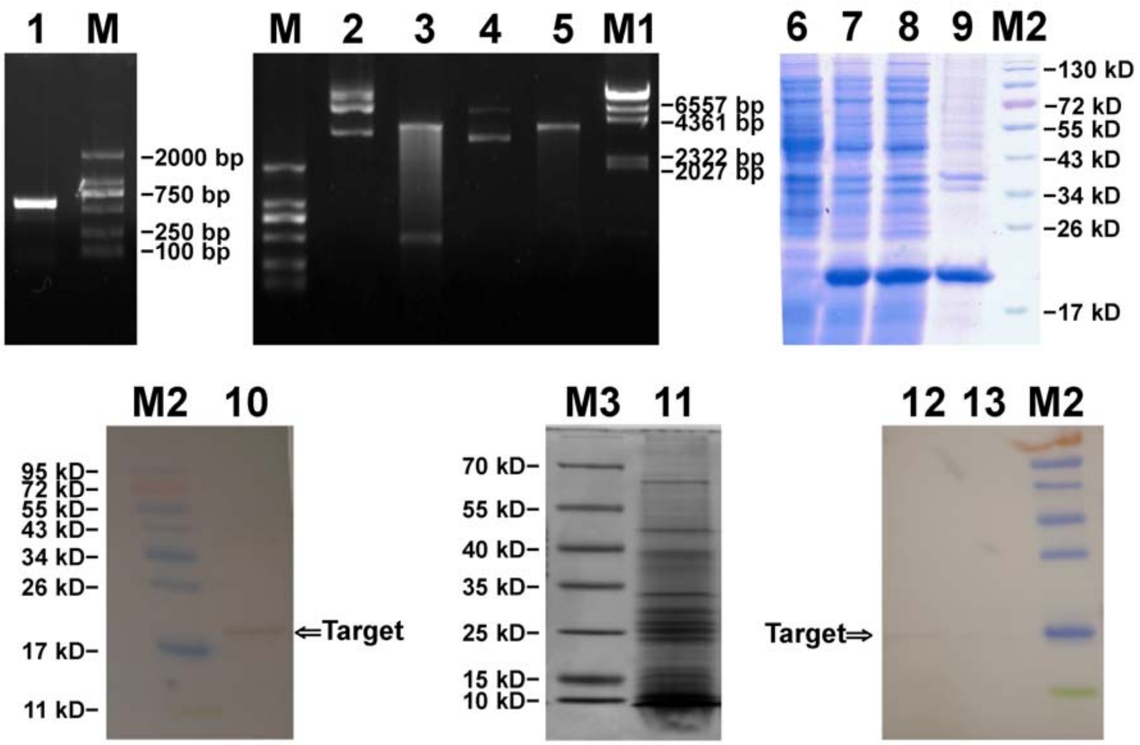

Figure S7. Electrophoresis patterns of the plasmid construction of pET-glnB, heterologous expression profile of MiPII in *Escherichia coli* (upper panel), purification of the recombinant MiPII, and specificity detection of its preparative polyclonal antibody by Western blot analysis in *Myrmecia incisa* (lower panel). Lane 1, amplified product of *MiglnB* ORF minus signal peptide sequence; Lane 2, recombinant plasmid pET-glnB; Lane 3, double digested products of plasmid pET-glnB with *Eco*RI and *Pst*I; Lane 4, plasmid pET-28a; Lane 5, double digested products of plasmid pET-28a; Lane 6, expression of recombinant protein MiPII without induction by addition of IPTG; Lane 7, MiPII expression induced by IPTG for 3 h; Lane 8, recombinant MiPII expressed in the supernatant of the transformant after sonication; Lane 9, recombinant MiPII expressed in the precipitate after sonication; Lane 10, Western blot analysis of recombinant MiPII with His-Tag antibody; Lane 11, total proteins isolated from *M. incisa*; Lanes 12 and 13, Western blot analysis of total proteins (approximately 25 μL and 10 μL, respectively) isolated from *M. incisa* with the purified MiPII polyclonal antibody; Lane M, DL 2000 DNA standard marker (Tiangen Biotech Co., Ltd.); Lane M1, DNA marker λDNA *Hind*III (Tiangen Biotech Co., Ltd.); Lane M2, prestained Protein ladder of standard protein (Fermentas); and Lane M3, ladder of standard protein (Fermentas).

Supplementary Figure S8

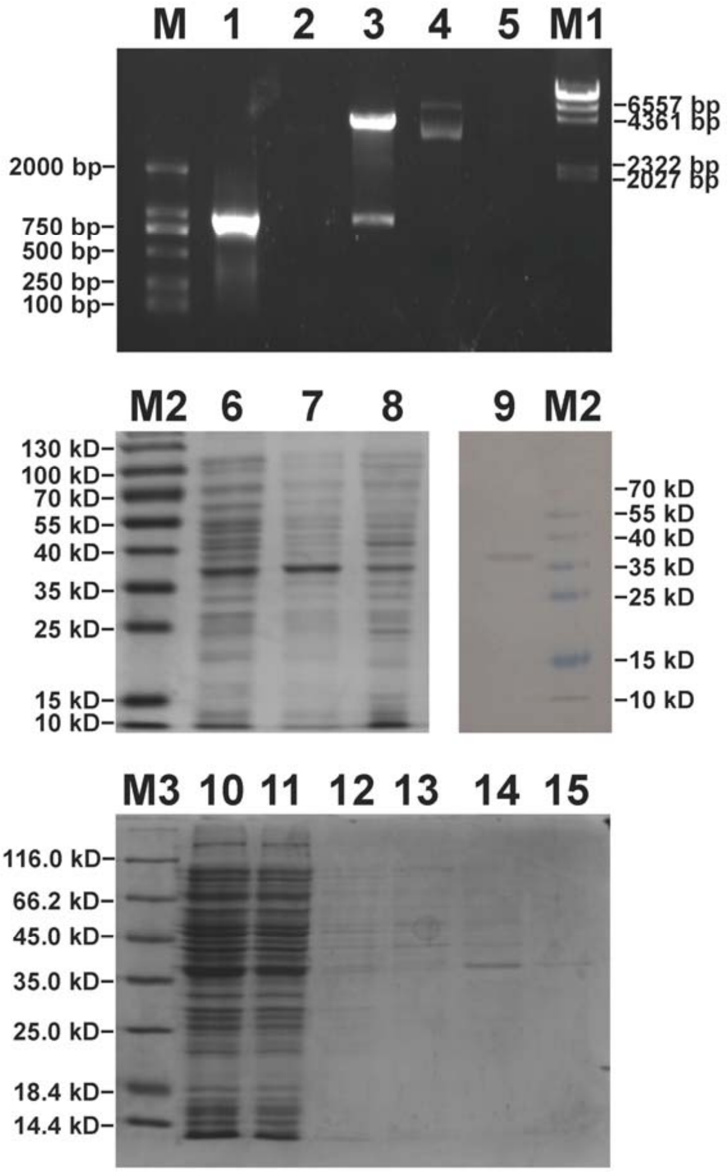

Figure S8. Recombinant expression profile and purification of MiNAGK from *Escherichia coli*.

Lane 1, amplified product of *MiargB* ORF minus signal peptide sequence; Lane 2, recombinant plasmid pET-argB; Lane 3, double digested products of plasmid pET-argB with *Eco*RI and *Xho*I; Lane 4, plasmid pET-28a; Lane 5: double digested products of plasmid pET-28a; Lanes 6, soluble crude extracts from the transformant; Lane 7, insoluble crude extracts from the transformant; Lane 8, whole bacterial cells; Lane 9, Western blot analysis of recombinant MiNAGK with His-Tag antibody; Lane 10, suspension of recombinant protein; Lane 11, elution of suspension of recombinant protein after Ni-NTA; Lanes from 12 through 15, elution of the suspension of recombinant protein by serial concentration of imidazole from 50, 150, 250, to 350 mM, respectively; Lane M, DL 2000 DNA standard marker (Tiangen Biotech Co., Ltd.); Lane M1, DNA marker λDNA *Hind*III (Tiangen Biotech Co., Ltd.); Lane M2, prestained Protein Ladder of standard protein (Fermentas); and Lane M3, ladder of standard protein (Fermentas).

Supplementary Figure S9

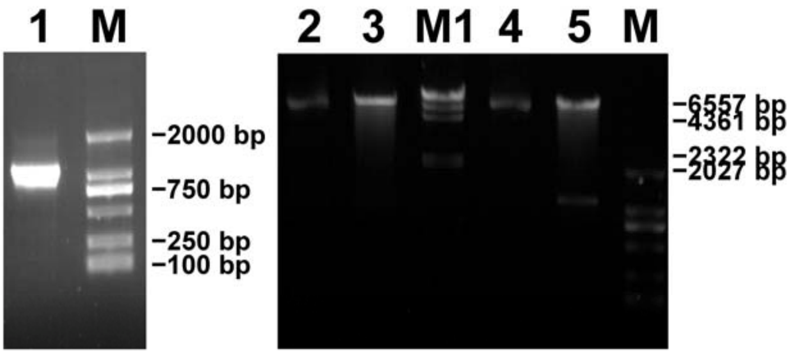

Figure S9. Agarose gel electropherogram of the binary vector pC-NAGK-GFP construction.

Lane 1, amplified product of *MiargB* ORF; Lane 2, recombinant plasmid pC-NAGK-GFP; Lane 3: double digested products of plasmid pC-NAGK-GFP; Lane 4, plasmid pC1300-GFP; 5: double digested products of plasmid pC1300-GFP; Lanes M and M1, the DL 2000 and λDNA *Hind*III, respectively, DNA standard markers.
